# Blocking axon-glial mechanotransduction to prevent concussive brain injury

**DOI:** 10.1186/s40478-025-02117-6

**Published:** 2025-09-29

**Authors:** Chao Sun, Di Ma, Jacob Hansen, Jeffrey R. Tonniges, Hongzhen Hu, Liwen Zhang, Chen Gu

**Affiliations:** 1 Molecular, Cellular and Developmental Biology graduate program, The Ohio State University, Columbus, OH 43210 USA; 2 Ohio State Biochemistry Program, The Ohio State University, Columbus, OH 43210 USA; 3 Department of Biological Chemistry and Pharmacology, The Ohio State University, 182 Rightmire Hall, 1060 Carmack Road, OH 43210 Columbus, USA; 4 Campus Microscopy and Imaging Facility, The Ohio State University, Columbus, OH 43210 USA; 5 Mark Lebwohl Center for Neuroinflammation and Sensation, Icahn School of Medicine at Mount Sinai, New York, NY 10029 USA; 6 Mass Spectrometry and Proteomics Facility, Comprehensive Cancer Center, The Ohio State University, Columbus, OH 43210 USA

## Abstract

All cells in the central nervous system (CNS) are considered mechanosensitive, but how they collectively respond to a concussive head impact and contribute to the transition from the primary to secondary injury remains unknown. Using a mouse model for mild traumatic brain injury (mTBI) or concussion, we report that blocking the activity of TRPV4 transient receptor potential channels inhibits mTBI-induced sequential changes of neurons and glial cells, as well as behavioral disturbances. A concussive head impact immediately induces axonal varicosities, preceding NMDA-receptor-mediated microglial activation and cortical demyelination. Afterward, these changes differentially and partially recover. Blocking TRPV4 channels before or after head impact markedly suppresses axon-glial and behavioral changes or enhances their recovery, respectively. Using knockout mice and AAV-Cre-mediated acute and cell-type-specific deletion, we further show that neuronal TRPV4 channels, as an mTBI target, regulate the homeostasis of axon mechanosensation and their hyperactivation causes axonal varicosity formation followed by axon-to-glia mechanotransduction.

**Supplementary Information:**

The online version contains supplementary material available at 10.1186/s40478-025-02117-6.

## Highlights

A concussive head impact induces axonal varicosities, preceding excitotoxicity-mediated cortical microglial activation and demyelination.

Blocking TRPV4 channels before or after head impact suppresses axon-glial and behavioral changes or enhances their recovery, respectively.

TRPV4 KO mice do not respond to TRPV4 blockers but remain vulnerable in mTBI, likely due to genetic compensation.

Acute deletion of neuronal TRPV4 inhibits mTBI-induced axonal varicosities and adjacent glial changes.

**Supplementary Information:**

The online version contains supplementary material available at 10.1186/s40478-025-02117-6.

## One sentence summary

Inhibition of axon-glial mechanotransduction through blocking TRPV4 channels prevents and mitigates concussive brain injury.

**Supplementary Information:**

The online version contains supplementary material available at 10.1186/s40478-025-02117-6.

## Introduction

Mild traumatic brain injury (mTBI) or concussion represents a major health problem worldwide. The mTBI is a diagnostic term for TBIs with loss of consciousness less than 30 min (min), an initial Glasgow Coma Scale of 13–15 after 30 min, and posttraumatic amnesia less than 24 h (h) [1]. Accurate diagnosis and effective treatment remain difficult because many aspects of mTBI are still poorly understood. For instance, it is unknown why most mTBIs recover, but approximately 15% of them develop long-term deficits [2–5]. The concussion threshold remains elusive. For instance, in studies of American football players, one of the most puzzling findings is that a given head impact that causes serious injury in some players often appears harmless for hundreds of other players [6]. In contrast to those with high concussion tolerance, a mild head impact can cause devastating injury in people with familial hemiplegic migraine (FHM) type 1, an autosomal dominant form of migraine with aura caused by gain-of-function mutations in the calcium channel gene (CACNA1A or Cav2.1) [7, 8]. Moreover, why different brain regions display different vulnerabilities to a head impact is still unclear [3, 9]. Importantly, the root of these problems is the question of which type of brain cells within the same brain region is most prone to mechanical injury in mTBI.

It is the current theme that all brain cells are mechanosensitive [10, 11]. Neurons and relatively softer glial cells, including microglia, astrocytes, and pre-myelinating oligodendrocytes, display differential viscoelastic properties that depend on their location in the brain, subcellular domains, and developmental and differentiation stages [12–16]. In mTBI, which usually does not involve bleeding, an external mechanical force likely creates larger deformation in glial cells than their adjacent neurons, but the deformation threshold leading to cellular injury in different cell types may differ and remains poorly understood. Furthermore, both neuronal and glial cell damages are involved in the secondary injury of mTBI, which shares some aspects of the secondary injury of moderate-to-severe TBIs, such as diffuse axonal injury, excitotoxicity, microglia-mediated inflammation, and demyelination [17–27]. Due to the lack of early biomarkers in mTBI, these interlinked secondary injuries are often independently measured days or even weeks after head impact(s) in various studies, leading to no identifiable causal relationship. Thus, it remains unknown whether neurons and glia within the same brain region simultaneously or sequentially respond to the mechanical stress of a concussive head impact.

Many types of mechanical-stress-induced injury, such as diffuse axonal injury, microglial activation, excitotoxicity, demyelination, cell death, and oxidative stress, appear to involve aberrant calcium increase in neurons and/or glia [28–33]. Among all the mechanosensitive and Ca^2+^-permeable ion channels that are expressed in these brain cells, the N-methyl-D-aspartate (NMDA) glutamate receptor could act as an initial sensor for a head impact, especially because it can be directly activated in vitro by mechanical stress in the absence of ligand [34, 35]. Indeed, a concussive head impact can quickly trigger glutamate-mediated excitotoxicity [36]. Excitotoxicity mediated by NMDA receptor hyperactivation has been implicated in synaptic alteration, microglial activation, and neuronal cell death in mTBI [30, 37, 38]. However, NMDA receptor antagonists failed in clinical trials for treating TBIs so far, likely resulting from their interference with the normal synaptic transmission [39]. Nonetheless, this important research direction still requires more extensive studies.

TRPV4 transient receptor potential channel represents another mechanosensitive Ca^2+^-permeable ion channel [34]. Gain-of-function missense mutations in the TRPV4 gene are linked to human diseases, including two major groups: autosomal dominant neuromuscular disorders (Charcot-Marie-Tooth disease type 2 C and distal spinal muscular atrophies) and skeletal disorders (skeletal dysplasias and osteoarthropathy) [40–43]. Our early studies implicated the TRPV4 channel in regulating mechanical stress-induced axonal varicosity formation in cultured central neurons, based on the results of ionic composition and Ca^2+^ influx, channel blockers and activator, RNAi knockdown, immunostaining, and reconstitution in HEK293 cells [28]. Mechanical stress-induced activation of TRPV4 channels leads to aberrant Ca^2+^ increase in axons, causing axonal varicosity formation via altering microtubule stability and hence axonal transport [28]. Axonal varicosities (swelling or beading) are enlarged, heterogeneous structures along axonal shafts that can be immediately induced by mechanical stress in vitro and in vivo [3, 28], and hence represent an early biomarker for axonal injury. However, the potential role of the TRPV4 channel in axonal varicosity formation in vivo is complicated by TRPV4’s broad expression in multiple types of brain cells, including neurons, microglia, oligodendrocyte progenitor cells, and endothelial cells [28, 44–47]. In particular, microglia were shown to be rapidly activated in brain injury as well [48, 49]. Thus, despite recent progress in structural understanding of TRPV4 channels [50–52] and the implication of TRPV4 in blood vessel damage in severe TBIs [53, 54], it remains unknown whether and how TRPV4 channel activation is involved in mTBI.

Using a mouse model of mTBI, namely the Closed-Head Impact Model of Engineered Rotational Acceleration (CHIMERA) [55], we show that a concussive head impact immediately induces axonal varicosities, followed by cortical microglial activation and demyelination. All these changes are partially reversible. Interestingly, whereas blocking NMDA receptor activity suppresses CHIMERA-induced microglial activation and cortical demyelination but not axonal varicosity formation, a TRPV4 channel blocker GSK2193874 (GSK219) suppresses the above-mentioned axon-glial changes, as well as behavioral disturbances. GSK219’s protective effects are absent in TRPV4 knockout (KO) mice, while the KO mice remain vulnerable to mTBI likely resulting from genetic compensation in axon mechanosensation. Using an acute and regional deletion strategy, as well as a different TRPV4 blocker, we have further demonstrated that the TRPV4 channel is a promising target for both preventing and mitigating mTBI.

## Results

### A concussive head impact induces sequential axon-glial changes with partial reversibility

**Fig. 1:**
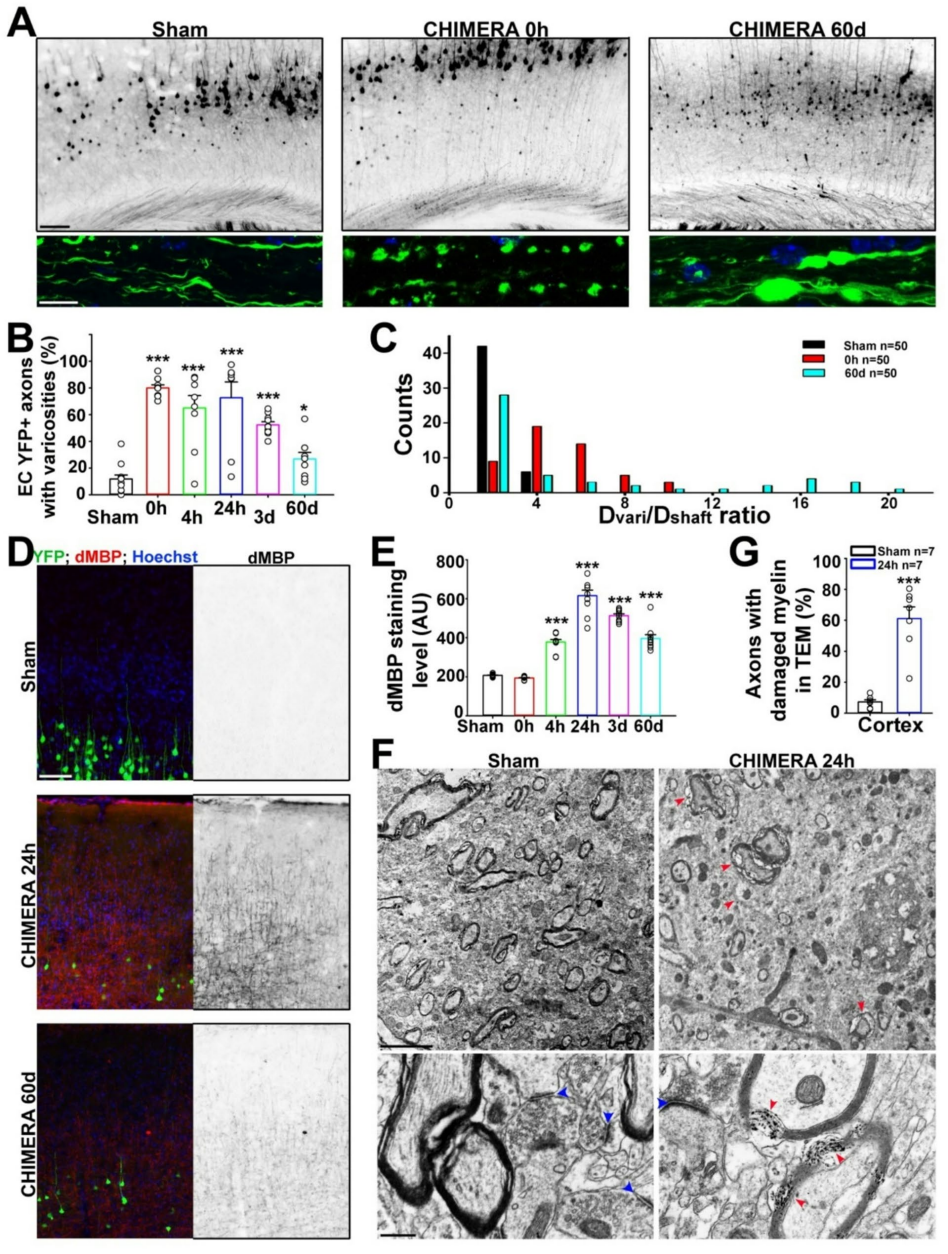
CHIMERA-induced axonal varicosities precede cortical demyelination with partial recovery. (**A**) YFP + cortical neurons from Thy1-YFP transgenic mice: no impact (Sham, left), 0 h (middle), or 60d (right) after one 0.9 J head impact in CHIMERA. YFP fluorescence signals are inverted in low-mag grayscale images (top) and are in green in high-mag confocal images from EC (bottom). (**B**) Summary of the percentage of YFP + axons with varicosities in EC at different time points after CHIMERA. (**C**) Size distribution of axonal varicosities (D_vari_/D_shaft_ ratio) in mice under three conditions, Sham, 0 h, and 60d. Image numbers: 50 in each condition. (**D**) Cortical demyelination caused by CHIMERA. YFP is in green, dMBP staining in red, and Hoechst in blue in merged images (left). The dMBP signals are inverted in grayscale images (right). (**E**) Summary of dMBP staining levels in the mouse cortex at different time points after CHIMERA. In (**B**) and (**E**), One-way ANOVA followed by Dunnett’s test: *** *p* < 0.001, * *p* < 0.05. Mouse numbers: 6 (Sham), 6 (0 h), 3 (4 h), 4 (24 h), 5 (3d), and 3 (60d). 1–3 images from each mouse were included. (**F**) TEM images from mouse cortex without head impact (Sham; left) or 24 h after CHIMERA (right). High-mag TEM images are at the bottom. Red arrowheads, damaged myelin sheath. Blue arrowheads, asymmetric synapses. (**G**) Summary of the percentage of axons with damaged myelin in the cortex, gray matter. Image numbers are provided. Unpaired t-test: *** *p* < 0.001. Scale bars, 250 μm in (**A**) (upper panels) and (**D**), 30 μm in (**A**)(lower panels), 2 μm and 300 nm in upper and lower panels in (**F**), respectively

To determine the fate of axonal varicosities induced by CHIMERA (0.9 J (0.9 J)), we visualized the YFP + axons in the brain of Thy1-YFP transgenic mice at different time points after a single head impact. Consistent with our recent results showing regional differences in axonal varicosity formation induced by a concussive head impact in mice [3], axonal varicosities were immediately (or 0 h (0 h) after impact) induced in the corpus callosum (CC), external capsule (EC), and the cortex. Then, the level of axonal varicosities remained high after 24 h (24 h) and declined after 3 days (3d) (Fig. 1A, B and Fig. S1). Two months (60d) after CHIMERA, the varicosity level became much lower than the peak level at 24 h, but was still significantly higher than that in sham in the CC and EC (Fig. 1A-C and Fig. S1). The reduction of axonal varicosity levels at later time points indicates some recovery in vivo, consistent with our in vitro and timelapse imaging results showing partial recovery of axonal varicosities after being induced by fluid mechanical stress [28]. Interestingly, we observed sparse but abnormally large axonal varicosities (3 ~ 5 fold larger diameter compared to the axonal varicosities induced at 0 h) in vivo at 60d post CHIMERA. These large and residual axonal varicosities were comparable to a neuronal cell body in size and not present at 0–24 h after CHIMERA (Fig. 1A, C). Taken together, the present study is the first to show changes in induced axonal varicosities at different time points after a concussive head impact.

Demyelination was reported across the spectrum of TBI severities, including mTBI in both human and animal models [19, 21, 24, 56, 57]. To determine temporal and spatial patterns of potential myelin alterations in CHIMERA, we first examined the immunostaining intensity of myelin basic protein (MBP), a reliable marker for mature myelin internodes. There was a significant reduction in the density of MBP + myelin internodes in the cortex at 24 h but not 0 h after head impact (Fig. S2). We further analyzed the myelin damage in the cortex using a dMBP antibody that recognizes degraded MBP molecules, and found that dMBP + signals in internodes started to increase at 4 h after impact, reached the peak level at 24 h, slowly declined after 3d, but did not return to the sham level even after 2 months (60d) (Fig. 1D, E).

To determine ultrastructural changes of myelin damage induced by CHIMERA, we performed transmission electron microscopy (TEM) on the cortex from sham mice or mice at 24 h after CHIMERA. At 24 h, axon myelination in the cortex was significantly reduced compared to the sham (Fig. 1F, G), consistent with the dMBP and MBP staining results. Different from the ones in white matter, the myelinated axons in gray matter (e.g. the cortex) were often observed with synapses nearby. Twenty-four hours (24 h) after CHIMERA, swelling presynaptic terminals with non-uniform synaptic vesicles were observed adjacent to axons with damaged myelin (Fig. 1F), raising a possible link between synaptic dysfunction and myelin damage in the cortex. Nonetheless, CHIMERA-induced myelin damage or demyelination in the cortex took place after axonal varicosity formation.

**Fig. 2:**
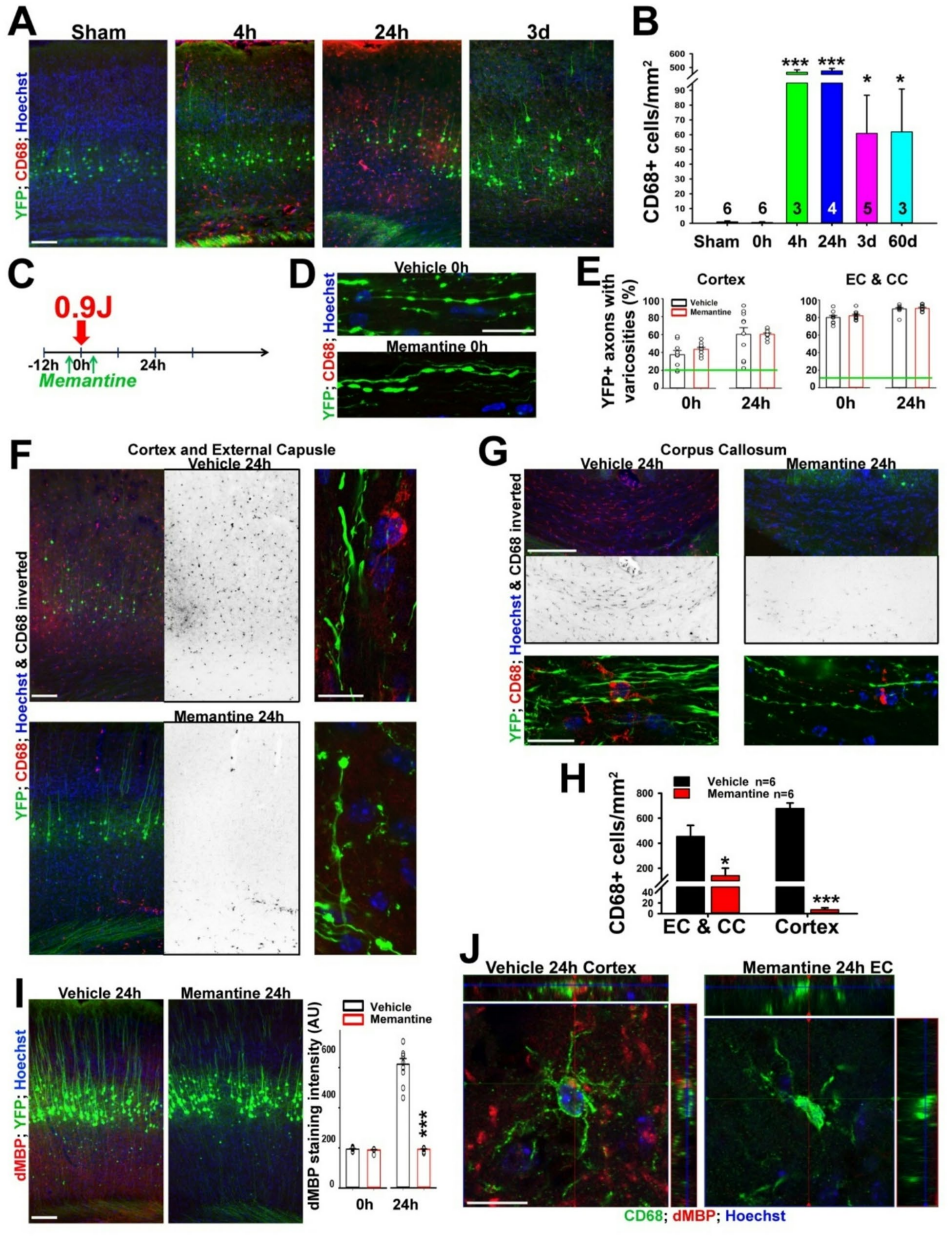
Memantine inhibits CHIMERA-induced microglial activation and cortical demyelination but not axonal varicosity formation. (**A**) CHIMERA-induced microglial activation in the cortex. Low-mag images of the cortex from mice received no impact (Sham, left), or 0 h, 4 h, 24 h, or 3d after head impact. The anti-CD68 staining signals are in red, YFP in green, and Hoechst in blue. (**B**) Summary of the density of CD68 + cells in the mouse cortex under different conditions. Mouse numbers are provided within the bars. 3–4 images from each mouse are included. One-way ANOVA followed by Dunnett’s test: * *p* < 0.05, *** *p* < 0.001. (**C**) Diagram for the memantine experiment. Memantine was injected (via i.p. at 10 mg/kg each dose) 1.5 h before CHIMERA and 3 h afterward. (**D**) Representative images of induced axonal varicosities at 0 h from vehicle (top) and memantine (bottom) treated Thy1-YFP transgenic mice. YFP fluorescence signals are in green, the CD68 signals in red, and Hoechst in blue. (**E**) Summary of the effect of memantine treatment on axonal varicosity induction in the cortex (left) and EC and CC (right) of Thy1-YFP transgenic mouse at 0 h and 24 h. Green lines, the basal levels in Sham. Unpaired t-test: no significant difference. *n* = 6 for all conditions. (**F**) Memantine treatment eliminated CD68 + cells in the cortex and significantly reduced CD68 staining signals in the EC 24 h after CHIMERA. CD68 staining signals are inverted in grayscale images. High-mag confocal images are on the right. (**G**) Memantine treatment markedly reduced CD68 + cells in the CC 24 h after CHIMERA. High-mag confocal images are at the bottom. (**H**) Summary of memantine’s effect on the density of CHIMERA-induced CD68 + cells at 24 h. Unpaired t-test: ** *p* < 0.01, *** *p* < 0.001. (**I**) Memantine treatment markedly reduced dMBP signals in the cortex 24 h after CHIMERA. Unpaired t-test: ***p < 0.001. *n* = 6 for all conditions. (**J**) Confocal image stacks of CD68 (green) and dMBP (red) costaining in the cortex of vehicle (left) and memantine-treated mice 24 h after CHIMERA. Scale bars, 250 μm in (**A**), (**F**) left, (**G**) upper, and (**I**); 25 μm in (**D**), (**F**) right, (**G**) lower, and (**J**)

Microglial activation has been implicated in axonal injury and demyelination in mTBI and other neurological disorders [58]. To determine its potential relationship with axonal varicosities and cortical demyelination induced by CHIMERA, we stained brain slices for activated microglia using an anti-CD68 antibody (labeling lysosomes in the soma of activated microglia) and performed fluorescence microscopy. CD68 + cell densities markedly increased 4 h after CHIMERA in the cortex, as well as in CC and EC, compared to those at 0 h and of sham; The CD68 + cell density and signal intensity sustained 24 h after CHIMERA, and significantly reduced but did not completely disappear after 3 days or 2 months (Fig. 2A, B). Our results are consistent with a recent meta-analysis for the activation time course of microglia in various mTBI animal models, in which the earliest time for microglial activation in white matter is 2 h after a concussive head impact [58]. The activated microglia, shown by CD68 staining signals, were distributed rather evenly throughout these brain regions.

Astrocytes represent the third major group of glial cells in the CNS. To determine whether CHIMERA (0.9 J) activates astrocytes, we performed the immunostaining using an anti-glial fibrillary acidic protein (GFAP) antibody, an astrocyte marker, on brain sections from Thy1-YFP mice at different time points. We focused on the hippocampus, CC, EC, and cortex, and found no clear morphological changes of GFAP + cells and no increase of GFAP expression (a marker for activated astrocytes) at all time points after head impact (Fig. S3). Thus, it is unlikely that CHIMERA (0.9 J) causes astrocyte activation. Moreover, an early comprehensive study showed that 0.9 J CHIMERA is unlikely to disrupt cerebrovasculature [59]. Taken together, we found that in CHIMERA (0.9 J), axonal varicosities were induced immediately after head impact (0 h), followed by microglial activation at 4 h and cortical demyelination at 24 h. The present study is the first to show such sequential axon-glial changes and partial recovery after mTBI.

### Memantine inhibits mTBI-induced microglial activation and cortical demyelination but not axonal varicosities

To identify a new strategy to inhibit CHIMERA-induced axonal and glial changes, we first examined memantine, a noncompetitive blocker for NMDA-type glutamate receptors to combat glutamate excitotoxicity, which was approved by the FDA for treating Alzheimer’s disease [60]. Memantine treatment was recently shown to inhibit synaptic changes and cognitive dysfunction in a mouse model for high-frequency subthreshold head impact [37]. This effect is consistent with the notion that NMDA receptor-mediated excitotoxicity is involved in mTBI [30, 37, 38]. Based on the pharmacokinetics of memantine from early studies [37], we treated the mice with the first dose of memantine 1.5 h before head impact and the second dose 3 h after head impact (Fig. 2C). The mice were perfused and fixed either 0 h (receiving only one dose of memantine) or 24 h after a head impact. Memantine pretreatment did not reduce the level of axonal varicosities induced by CHIMERA in the cortex, CC, or EC (Fig. 2C-E).

Interestingly, memantine treatment markedly suppressed microglial activation in both the cortex and white matter CC and EC 24 h after head impact (Fig. 2F-H). When mice were pretreated with memantine, whereas CD68 + cells were almost completely absent across different cortical layers, a low level of CD68 + cells was still observed in the CC and EC at 24 h after CHIMERA (Fig. 2F-H). Thus, while microglia in the cortical gray matter appeared exclusively activated by NMDA-receptor-mediated excitotoxicity, microglia in the EC and CC (white matter) could be partially activated by damage-associated molecular patterns (DAMPs) that are independent of NMDA receptors, such as ATP, heat shock proteins, and high mobility group box 1 protein [61]. Furthermore, we found that cortical demyelination at 24 h revealed by the dMBP staining, was eliminated by memantine treatment (Fig. 2I). Confocal images showed that CD68 + microglial processes and dMBP + myelin internodes did not extensively colocalize (Fig. 2J). Taken together, these results suggest that whereas CHIMERA-induced microglial activation and cortical demyelination are mainly mediated by NMDA receptor activation, axonal varicosity induction does not require NMDA receptor activation, which is consistent with our results that excitotoxicity did not acutely induce axonal varicosities in cultured central neurons [28].

### GSK2193874 (GSK219) markedly inhibits mTBI-induced axon-glial and behavioral alterations

**Fig. 3:**
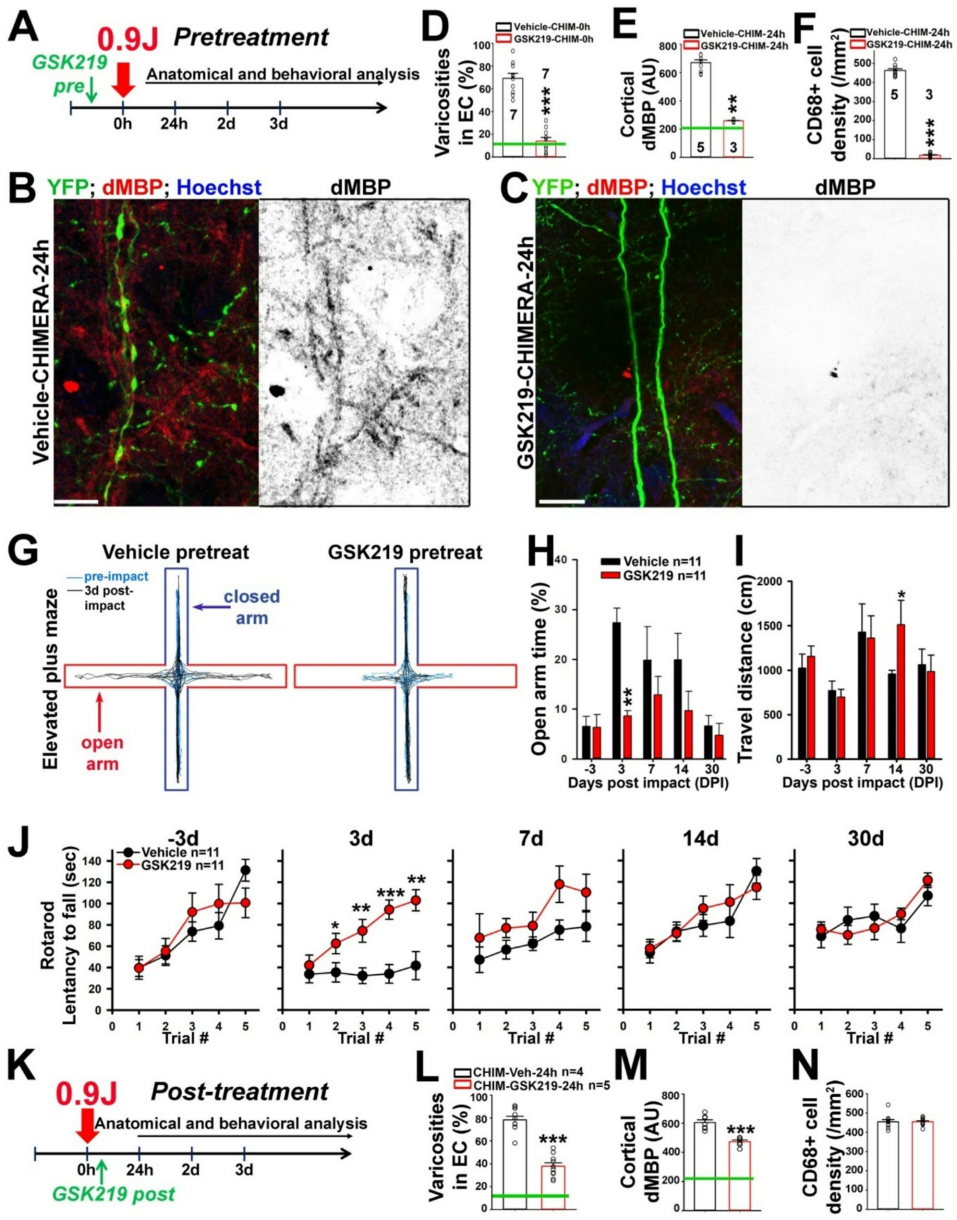
GSK219 markedly inhibits CHIMERA-induced axonal varicosities, microglial activation, cortical demyelination, and behavioral alterations. (**A**) Diagram for the CHIMERA experiment with GSK219 pretreatment. Mice were gavaged with GSK219 (20 mg/kg) or vehicle (as control) once 3 h before CHIMERA (0.9 J). (**B**)-(**C**) Confocal images of the cortex of Thy1-YFP mice 24 h after CHIMERA with the pretreatment of vehicle (**B**) or GSK219 (**C**). The dMBP staining signals are inverted in grayscale images (right) and in red in merged images (left), YFP in green, and Hoechst in blue. (**D**) Summary of YFP + axons with varicosities in EC 0 h after CHIMERA with vehicle or GSK219 pretreatment. The green line, the basal level in Sham. The mouse numbers are provided in the chart. 1–3 images from each mouse are included. Unpaired t-test: *p* = 0.0000021. (**E**) Summary of dMBP intensity in the cortex 24 h after CHIMERA with vehicle or GSK219 pretreatment. The green line, the basal level in Sham. Unpaired t-test: *p* = 7.481 × 10^− 12^. (**F**) Summary of CD68 + cell density in the cortex 24 h after CHIMERA with vehicle or GSP219 pretreatment. The basal level in Sham is close to 0. Unpaired t-test: *p* = 3.257 × 10^− 14^. (**G**) Example traces of mouse movement in the elevated plus maze (EPM) before (cyan) or 3d after CHIMERA (black) with vehicle (left) or GSK219 pretreatment (right). (**H**) Summary of the percentage time of open arms, 100 × T_open_/(T_open_+T_closed_), in the EPM test. Unpaired t-test: *p* = 0.00408 at 3 DPI. (**I**) Summary of the total travel distance in the EPM test. Unpaired t-test: *p* = 0.0498 at 14 DPI. (**J**) Rotarod results before CHIMERA (-3d), and 3d, 7d, 14d, and 30d after CHIMERA. Unpaired t-test in 3d: trial 2 *p* = 0.0157; trial 3 *p* = 0.00606; trial 4 *p* = 0.000829; trial 5 *p* = 0.00133. (**K**) Diagram for the CHIMERA experiment with GSK219 post-treatment. Mice were gavaged with GSK219 (20 mg/kg) or vehicle (as control) once 2 h after CHIMERA (0.9 J). (**L**) Summary of YFP + axons with varicosities in EC 24 h after CHIMERA with vehicle or GSK219 post-treatment. The green line, the basal level in Sham. Unpaired t-test: *p* = 0.000205. (**M**) Summary of dMBP intensity in the cortex 24 h after CHIMERA with vehicle or GSK219 post-treatment. The green line, the basal level in Sham. Unpaired t-test: *p* = 0.0000517. (**N**) Summary of CD68 + cell density in the cortex 24 h after CHIMERA with vehicle or GSK219 post-treatment. The basal level in Sham is close to 0. *p* = 0.957. Unpaired t-test: * *p* < 0.05, ** *p* < 0.01, *** *p* < 0.001. Scale bars, 20 μm in (**B**) and (**C**)

Our recent mechanistic studies indicated that TRPV4 plays a key role in axonal varicosity induction by mechanical stress in cultured CNS neurons [28], but how TRPV4 regulates mTBI-induced axonal varicosities in vivo remains unknown. To determine whether blocking TRPV4 channel activity can inhibit CHIMERA-induced axonal varicosities, we first performed the pretreatment experiment using TRPV4 channel blocker GSK219 in CHIMERA (0.9 J). GSK219 was shown orally active and highly selective for the TRPV4 channel, and its half-life in the body is approximately 10 h [62]. A single dose of GSK219 through gavage 3 h before the impact markedly suppressed axonal varicosity formation in the cortex, CC, and EC 0 h and 24 h after CHIMERA (Fig. 3A-D). Interestingly, the GSK219 pretreatment also completely inhibited microglial activation and cortical demyelination as revealed by CD68 and dMBP staining, respectively, in these brain regions (Fig. 3B, C, E, F and Fig. S4A, B). As a negative control, vehicle gavage did not provide any protection. It was reported that TRPV4 regulates hypotonic morphological changes of retinal microglia and temperature-dependent motility of microglia in the brain [63–65]. TRPV4 activity also regulates the proliferation of oligodendrocyte precursor cells (OPCs) but not their differentiation into mature oligodendrocytes [47]. Thus, our results suggest that GSK219 may block TRPV4 channels in neurons to protect the brain from mTBI, but do not rule out its potential action on TRPV4 channels in glial cells.

To determine how GSK219 pretreatment affects behavioral alterations induced by 0.9 J CHIMERA, we performed a battery of behavioral assays as we recently published [66]. Reduced risk avoidance in the elevated plus maze (EPM) was caused by CHIMERA for up to 14 days, and GSK219 pretreatment suppressed the avoidance reduction (Fig. 3G-I). In the rotarod, CHIMERA caused a marked worsening of motor coordination and activity at 3d and 7d but largely recovered after 14d, whereas the GSK219 pretreatment significantly improved the rotarod performance at 3d (Fig. 3J) and hence prevented poor performance in rotarod caused by CHIMERA. In the balance beam test, the motor balance was markedly impaired at 7d after CHIMERA and recovered at 14d, whereas the GSK219 pretreatment significantly improved the balance beam performance at 7d (Fig. S4C). In the open-field test, the total travel distance significantly decreased after CHIMERA, and moderately increased afterward, whereas the GSK219 pretreatment had no effect (Fig. S4D). In the novel object recognition (NOR) test, CHIMERA reduced the recognition memory 3d after CHIMERA, and this effect persisted, whereas the GSK219 pretreatment reversed the recognition memory deficit at 30d (Fig. S4E). In the Y-maze test, CHIMERA did not change spontaneous alternation, and the GSK219 pretreatment had no effect (Fig. S4F). Taken together, TRPV4 inhibition before the head impact almost completely suppressed axon-glial and behavioral changes in CHIMERA.

To determine how GSK219 posttreatment might mitigate mTBI, we gavaged the mice with a single dose of GSK219 3 h after CHIMERA. Twenty-four hours (24 h) after head impact, mice with GSK219 posttreatment displayed fewer axonal varicosities compared to vehicle treatment at 24 h, as well as reduced cortical demyelination (Fig. 3K-M and Fig. S5A-D). GSK219 posttreatment did not inhibit microglial activation at 24 h (Fig. 3N and Fig. S5E). GSK219 posttreatment did not reduce time spent in the open arms of EPM at all time points but did significantly increase the latency to fall in the rotarod 20d post-impact and decrease the time to cross a balance beam, suggesting that the posttreatment improved motor coordination, balance, and mobility (Fig. S5F-H). GSK219 posttreatment did not change the results in the open-field, NOR, and Y maze tests. Therefore, blocking TRPV4 activation after a concussive head impact mitigates mTBI, likely through enhancing the recovery of axonal injury and some behavioral disturbances.

Since GSK219 has striking beneficial effects in protecting and mitigating brain injuries induced by CHIMERA (0.9 J), it is of paramount importance to verify its target(s). Although GSK219 is highly selective to the TRPV4 channel (IC50 = 48 nM), GSK219 can target other ion channels as well, with less efficacy, for instance, hERG or Kv11.1 (IC50 = 2300 nM), and L-type Cav1.2 (IC50 = 5900 nM) [62]. Nonetheless, it is still important to address the potential off-target effect of GSK219.

### TRPV4 KO mice do not respond to GSK219 and remain vulnerable in mTBI

**Fig. 4:**
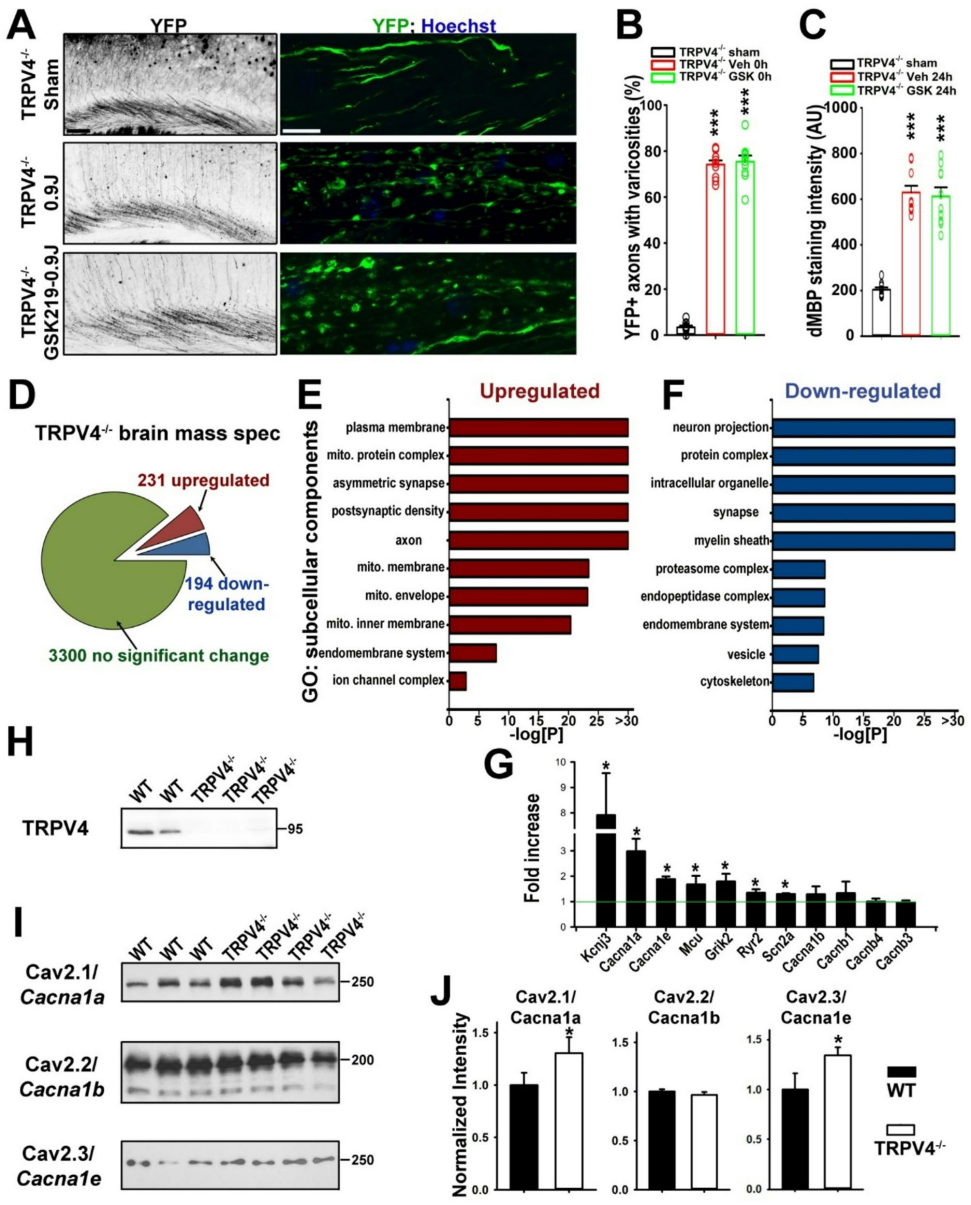
Protective effects of GSK219 are absent in TRPV4^−/−^ mice after head impact in CHIMERA. (**A**) Low-mag (left) and confocal (right) images from the cortex of TRPV4^−/−^ mice received no impact (Sham, top), or 0 h after CHIMERA (0.9 J) with vehicle (middle) or GSK219 pretreatment (bottom). YFP signals are inverted in gray-scale images and green in confocal images. Scale bars, 200 μm in left panels, and 20 μm in right panels. (**B**) Summary of the effects of TRPV4 KO and GSK219 pretreatment on axonal varicosity formation at 0 h after CHIMERA. (**C**) Summary of the effects of TRPV4 KO and GSK219 pretreatment on dMBP staining intensities in the cortex 24 h after CHIMERA. In (**B**) and (**C**), One-way ANOVA followed by Dunnett’s test: *** *p* < 0.001. Mouse numbers: 4 (Sham), 5 (Veh 0 h), and 4 (GSK 0 h). 1–3 images from each mouse are included. (**D**) Protein changes in the brains of TRPV4^−/−^ mice versus age- and sex-matched WT mice revealed by genome-wide proteomics with mass spectrometry analysis. (**E**) Upregulated proteins in the TRPV4^−/−^ mouse brain in GO subgroups based on subcellular components. (**F**) Down-regulated proteins in the TRPV4^−/−^ mouse brain in GO subgroups. (**G**) The upregulated ion channel proteins versus unchanged Cav channels. Unpaired t-test; Kcnj3: *p = 0.0075; Cacna1a: *p = 0.0024; Cacna1e: *p = 0.011; Mcu: *p = 0.0049 ; Grik2: *p = 0.038; Ryr2: *p = 0.033; Scn2a: *p = 0.027. (**H**) TRPV4 deletion was verified by Western blotting with WT and TRPV4^−/−^ brains. (**I**) Western blots of WT and TRPV4^−/−^ brains using antibodies against 3 Cav channel proteins. Numbers on the right, molecular weights in kDa. (**J**) Quantification summary of Western blot results. Unpaired t-test: * *p* < 0.05

To determine whether GSK219’s beneficial effects on mTBI were indeed through TRPV4 channels, we performed the GSK219 pretreatment in CHIMERA using TRPV4 global KO (TRPV4^−/−^) mice. We crossed TPRV4^−/−^ mice with Thy1-YFP transgenic mice to label the axons of a subset of projection neurons, to generate TRPV4^−/−^;Thy1-YFP mice. In sham, there was only the basal level of axonal varicosities in the background of TRPV4^−/−^, similar to that of WT mice. Markedly increased levels of axonal varicosities (0 h) and cortical demyelination (24 h) were found after CHIMERA in TRPV4^−/−^;Thy1-YFP mice either pretreated with vehicle or GSK219 (Fig. 4A-C). Therefore, the GSK219 pretreatment failed to prevent CHIMERA-induced axon-glial changes in TPRV4^−/−^ mice, confirming that GSK219’s effects on WT mice in CHIMERA were indeed mediated by TRPV4 channels.

It is important to note that the TRPV4 global deletion did not render significant mTBI resistance in 0.9 J CHIMERA. Axonal varicosity formation and cortical demyelination were still clearly found in TRPV4^−/−^ mice after CHIMERA, similar to WT mice. We hypothesized that there might be a compensatory mechanism during development in the absence of TRPV4 channels. To assess potential changes other than the TRPV4 protein in the brain of TRPV4^−/−^ mice, we performed a genome-wide mass spectrometry analysis using brains from TRPV4^−/−^ and their age- and sex-matched WT mice. Among 3725 proteins that were detected, 231 proteins were upregulated and 194 were downregulated with statistical significance, while the rest 3300 proteins had no significant change (Fig. 4D). Among upregulated proteins, the gene ontology (GO) analysis showed that the top two changes were from the proteins associated with the plasma membrane and mitochondria (Fig. 4E and Fig. S6). Among the down-regulated proteins, the GO analysis revealed different groups of proteins (Fig. 4F). Among upregulated Ca^2+^-permeable ion channels, two voltage-gated Ca^2+^ (Cav) channels, Cav2.1 (P/Q-type) and Cav2.3 (R-type) were at the top (Fig. 4G). TRPV4 proteins were not detected in mass spectrometry, but the Western blotting results verified the deletion of TPRV4 proteins (Fig. 4H and Fig. S7A). To verify the results from mass spectrometry, we performed the Western blot analysis for three Cav2 channels. Indeed, Cav2.1 and Cav2.3, but not Cav2.2, were significantly upregulated as revealed by Western blotting (Fig. 4I, J and Fig. S7B-D). Our immunostaining results of brain slides from WT and TRPV4^−/−^ mice revealed upregulation of Cav2.1 (Fig. S8). Thus, there are indeed many changes besides the deletion of the TRPV4 gene in the TRPV4^−/−^ mouse brain, which may be responsible for TRPV4-independent mechanosensation in neurons and/or glial cells.

### Acute deletion of neuronal TRPV4 inhibits mTBI-induced axonal varicosities and adjacent glial changes

**Fig. 5:**
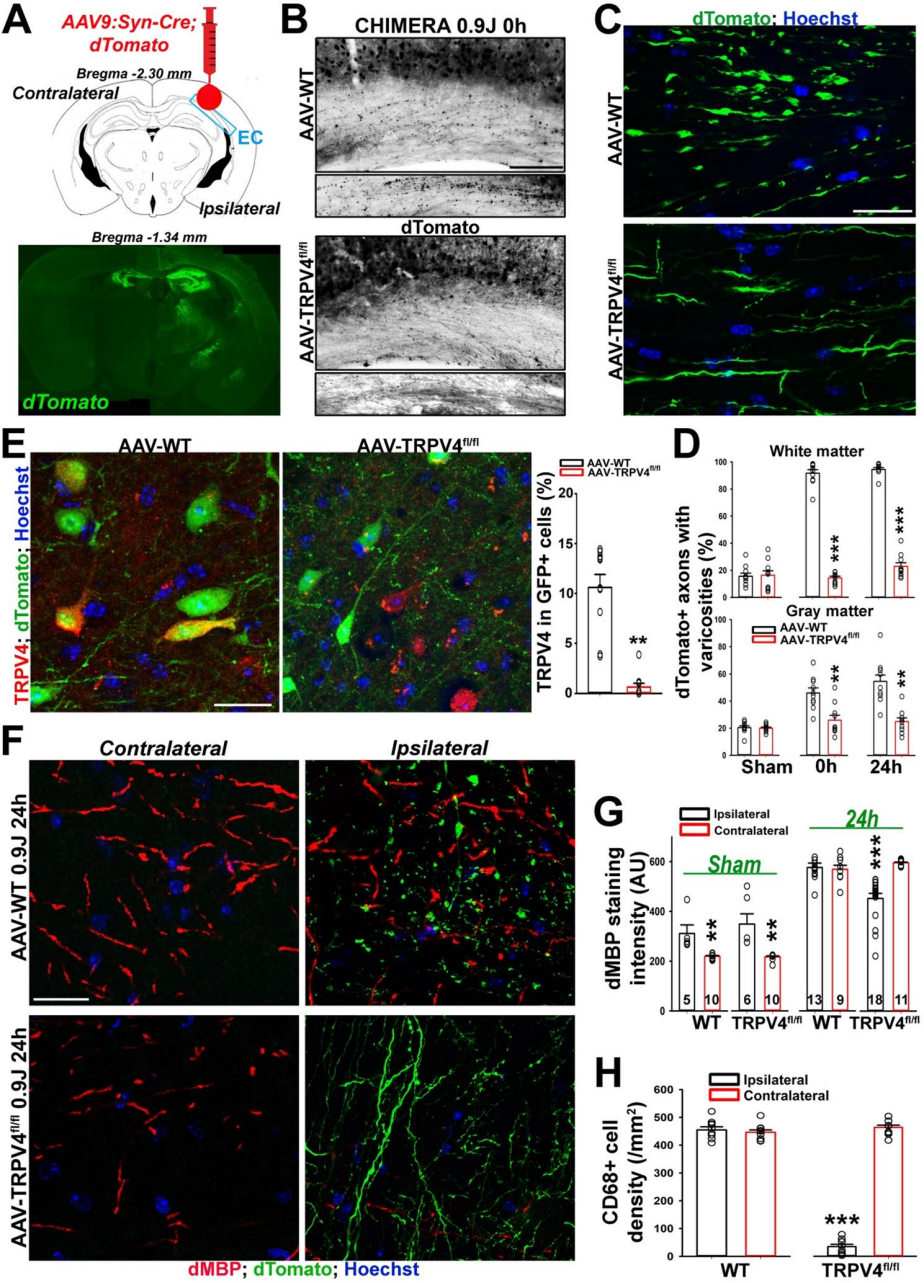
Acute deletion of neuronal TRPV4 prevents CHIMERA-induced axonal varicosity formation and adjacent glial changes. (**A**) Diagram of injecting AAV9-hSyn-Cre-dTomato into the right cortex of mice. Adult WT and TRPV4^fl/fl^ mice were used in CHIMERA about 1 month after the viral injection. A low-mag image (bottom) shows the injection sites with dTomato fluorescence in green and Hoechst in blue. (**B**) Low-mag gray-scale images with dTomato signals inverted of the cortex and EC from a WT (top) or a TRPV4^fl/fl^ (bottom) mouse injected with AAV9:Syn-Cre; dTomato at 0 h after CHIMERA. (**C**) Confocal images from the EC of the mice in (**B**). The dTomato signals are in green and Hoechst in blue. (**D**) Summary of the percentage of dTomato + axons with varicosities in white matter (CC and EC) and gray matter (cortical layers 1–6) from WT (black) and TRPV4^fl/fl^ (red) mice 0–24 h after CHIMERA, compared to Sham. *n* = 7 for all conditions. (**E**) Confocal images and quantification of TRPV4 protein (red) knockdown via the infection by AAV9-Syn-Cre-dTomato (green) in the cortex of TRPV4^fl/fl^ but not WT mice. Unpaired t-test: **, *p* < 0.01. *n* = 7 for both conditions. (**F**) Confocal images from the Ipsilateral and Contralateral sides of the cortex from WT (top) or TRPV4^fl/fl^ (bottom) mice 24 h after CHIMERA. The dTomato signals are in green and dMBP signals are in red. (**G**) Summary of dMBP staining intensity from the mouse cortex under different conditions. (**H**) Summary of CD68 + cell density in the cortex from WT or TRPV4^fl/fl^ (Flox) mice with AAV injection 24 h after CHIMERA. *n* = 10 in all groups. Unpaired t-test: **, *p* < 0.01; ***, *p* < 0.001. Scale bars, 160 μm in (**B**); 40 μm in (**C**), (**E**), and (**F**)

To determine whether acute deletion of neuronal TRPV4 channels can prevent axonal varicosity formation and glial changes in CHIMERA, we injected AAV9-hSyn-Cre-dTomato into the right cortex of TRPV4^fl/fl^ mice to induce the conditional deletion of TRPV4 channels in infected neurons, or into the right cortex of WT mice as a control. Four weeks (28d) after injection, these mice were used in CHIMERA (0.9 J) (Fig. S9A). Immediately (0 h) or 24 h after head impact, axonal varicosity induction was significantly reduced in dTomato + axons from TRPV4^fl/fl^ but not WT mouse brains (Fig. 5A-D). The deletion of TRPV4 channel proteins was effective, as shown in the comparison of anti-TRPV4 staining for AAV-WT and AAV-TRPV4^fl/fl^ injected with AAV9-hSyn-Cre-dTomato (Fig. 5E). Importantly, 24 h after CHIMERA, there was a significantly lower intensity of dMBP staining in the cortex of the injection side (ipsilateral), compared to the contralateral side in TRPV4^fl/fl^ mice, and this effect was absent in WT mice (Fig. 5F, G). This result indicates that dMBP upregulation as a marker of demyelination is tightly linked to axonal varicosity formation. We further examined microglial activation using the anti-CD68 antibody and found significantly reduced CD68 + cells in the ipsilateral side of the cortex with many AAV-infected and dTomato + neurons 24 h after CHIMERA in TRPV4^fl/fl^ but not WT mice (Fig. 5H and Fig. S9). There were more dMBP staining signals on the ipsilateral (injected) side compared to the contralateral (uninjected) side in the sham (Fig. 5G). These dMBP signals were mainly located around the injection path and thus likely caused by the microinjury from the injecting needle during AAV injection. Taken together, these results show that acute deletion of neuronal TRPV4 inhibits CHIMERA-induced axonal varicosities, which further leads to the inhibition of adjacent microglial activation and cortical demyelination.

### A different TRPV4 channel blocker, GSK2798745 (GSK279), inhibits axon-glial mechanotranduction in WT but not TRPV4^−/−^ mice

**Fig. 6:**
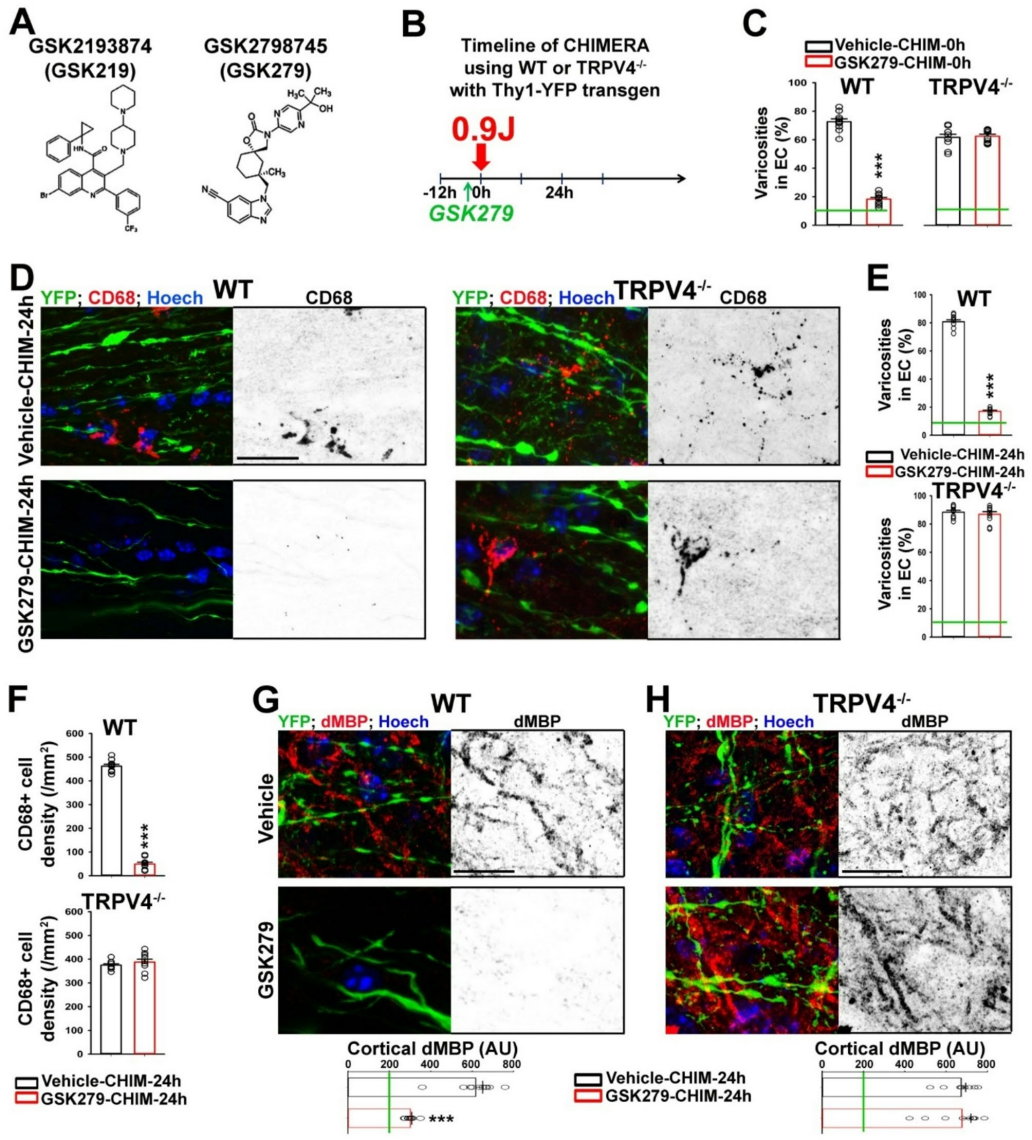
GSK279 markedly inhibits CHIMERA-induced axon-glial changes in WT but not TRPV4^−/−^ mice. (**A**) Structural diagrams of GSK219 and GSK279. (**B**) Diagram for the CHIMERA experiment with GSK279 pretreatment. WT; Thy1-YFP or TRPV4^−/−^;Thy1-YFP mice (3–4 months old) were injected via tail vein with GSK279 (18 µg/kg) or vehicle (as control) once 1.5 h before CHIMERA (0.9 J). Mice were perfused and fixed either immediately (0 h) or 24 h after head impact. (**C**) Summary of YFP + axons with varicosities in EC 0 h after CHIMERA with vehicle or GSK279 pretreatment. The green line, the basal level in Sham. (**D**) Confocal images of the EC of WT (left) and TRPV4^−/−^ (right) mice 24 h after CHIMERA with the pretreatment of vehicle (top) or GSK279 (bottom). The CD68 staining signals are inverted in grayscale images (right) and red in merged images (left), YFP in green, and Hoechst in blue. (**E**) Summary of YFP + axons with varicosities in EC 24 h after CHIMERA. (**F**) Summary of CD68 + cell density in the cortex 24 h after CHIMERA. (**G**) The dMBP staining signals (inverted in gray-scale images (right) and in red in merged images (left) in the cortex of WT mice 24 h after CHIMERA with vehicle (top) or GSP279 (middle) pretreatment. The summary is at the bottom. (**H**) The dMBP staining signals in the cortex of TRPV4^−/−^ mice 24 h after CHIMERA with vehicle or GSK279 pretreatment. Mouse numbers, *n* = 3 for all groups. 1–3 images from each mouse are included. Unpaired t-test: ***, *p* < 0.001. Scale bars, 20 μm in (**D**), (**G**), and (**H**)

To further verify the role of TPRV4 channel activity in axon-glial mechanotransduction, we examined another blocker, GSK279 [67], which structurally differs from GSK219 (Fig. 6A). We injected GSK279 into the tail vein of Thy1-YFP transgenic mice on the background of WT or TRPV4^−/−^ 1.5 h before CHIMERA (0.9 J). Mice were perfused and fixed either immediately (0 h) or 24 h after head impact (Fig. 6B). The GSK279 pretreatment markedly decreased the levels of axonal varicosities 0–24 h after CHIMERA in WT but not TRPV4^−/−^ mouse brains (Fig. 6C-E). Twenty-four hours after CHIMERA, GSK279 pretreatment markedly reduced microglial activation and demyelination, reflected by the staining intensities of CD68 and dMBP in the cortex, EC, and CC of WT but not TRPV4^−/−^ mouse brains (Fig. 6D, F-H). Therefore, similar to the effects of GSK219 pretreatment (Figs. 3A-F and 4A-C), GSK279 pretreatment also significantly reduced CHIMERA-induced axon-glial changes in WT mouse brains, whereas the protective effects were absent in TRPV4^−/−^ mouse brains. Taken together, our findings provide compelling evidence supporting the key role of TRPV4 in the axon-glial mechanosensation of the CNS in mTBI.

### Gabapentin significantly inhibits axon-glial mechanotransduction in the absence of TRPV4

To assess the possibility of genetic compensation for the absence of TRPV4 in axon mechanosensation, we further examined Cav channels (Cav2.1 and Cav2.3) that were upregulated in the TRPV4^−/−^ brain identified by mass spectrometry analysis (Fig. 4D-J). In particular, Cav2.1 is an interesting lead because its gain-of-function mutations were linked to FHM1 in humans [68, 69]. People with FHM1 have significantly increased susceptibility to mTBI [7, 8]. Thus, it is plausible that upregulated Cav2.1 channels in the absence of TRPV4 may mediate axon mechanosensation. However, the established specific blockers for Cav2.1 channels are toxin peptides and are unlikely to efficiently cross the blood-brain barrier. Here, we focus on gabapentin, which is a ligand of the α_2_δ subunit of Cav channels [70, 71]. The α_2_δ is an auxiliary subunit binding the main α_1_ subunit (the channel-forming protein) of high voltage-activated Cav channels, including not only Cav2.1 (P/Q-type) and Cav2.3 (R-type), but also Cav1 (L-type) and Ca2.2 (N-type) [71]. The α_2_δ subunit regulates Cav channel trafficking and biophysical properties [71]. Gabapentin is an FDA-approved drug to treat seizures, nerve pain, and restless legs syndrome, despite some side effects, including respiratory depression [72, 73].

**Fig. 7:**
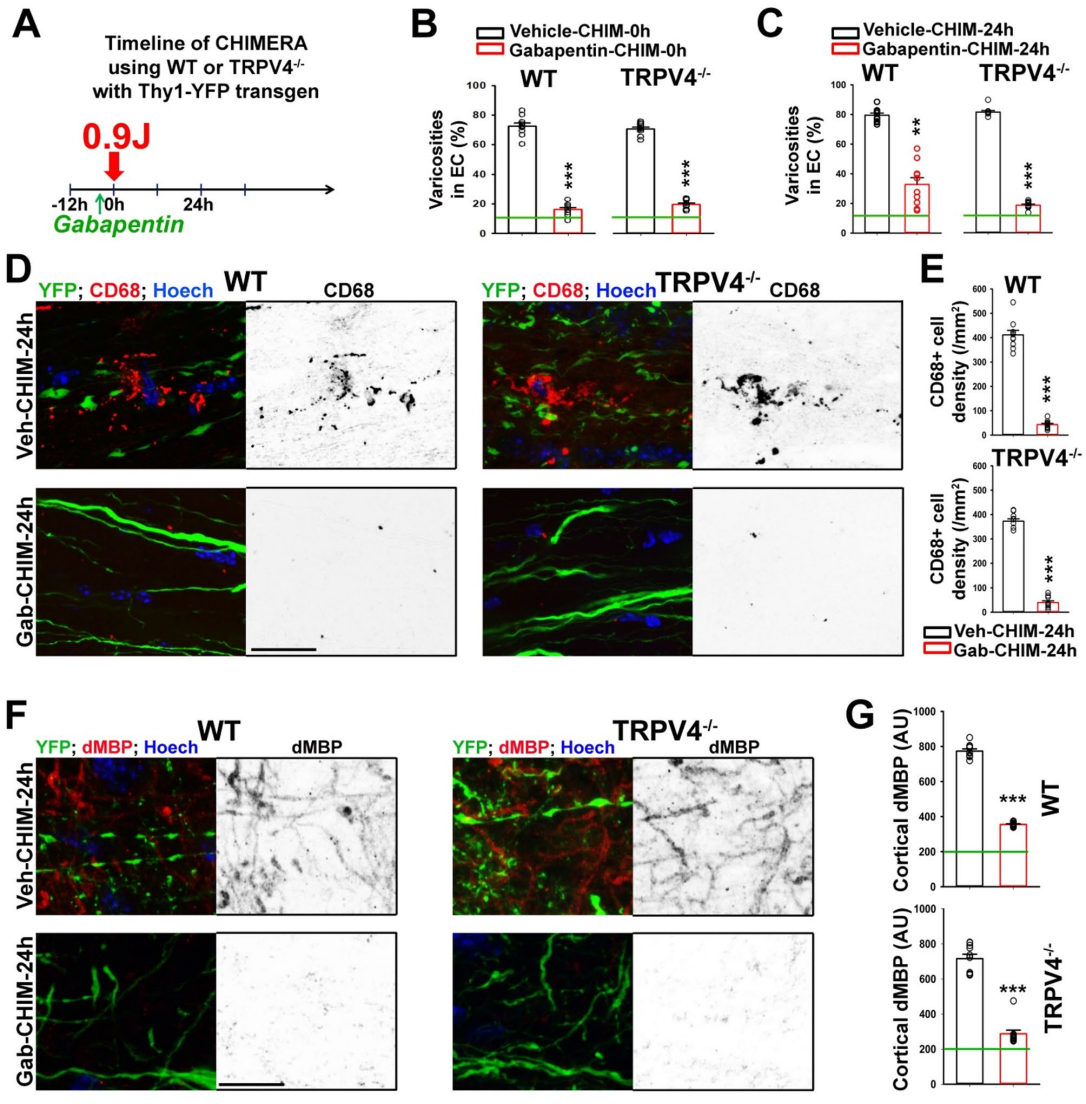
Gabapentin markedly inhibits CHIMERA-induced axon-glial changes in TRPV4^−/−^ mice. (**A**) Diagram for the CHIMERA experiment with gabapentin pretreatment. WT; Thy1-YFP or TRPV4^−/−^;Thy1-YFP mice were injected via I.P. with gabapentin (50 mg/kg) or vehicle (as control) once 1.5 h before CHIMERA (0.9 J). Mice were perfused and fixed either immediately (0 h) or 24 h after head impact. (**B**) Summary of YFP + axons with varicosities in EC 0 h after CHIMERA with vehicle or gabapentin pretreatment. The green line, the basal level in Sham. (**C**) Summary of YFP + axons with varicosities in EC 24 h after CHIMERA. (**D**) Confocal images of the EC of WT (left) and TRPV4^−/−^ (right) mice 24 h after CHIMERA with the pretreatment of vehicle (Veh; top) or gabapentin (Gab; bottom). The CD68 staining signals are inverted in grayscale images (right) and in red in merged images (left), YFP in green, and Hoechst in blue. (**E**) Summary of CD68 + cell density in the cortex 24 h after CHIMERA. (**F**) The dMBP staining signals in the cortex of WT (left) or TRPV4^−/−^ (right) mice 24 h after CHIMERA. (**G**) Summary of dMBP signals in the cortex 24 h after CHIMERA. Mouse numbers, *n* = 3 for all groups. 1–3 images from each mouse are included. Unpaired t-test: **, *p* < 0.01; ***, *p* < 0.001. Scale bars, 25 μm in (**D**) and (**F**)

To determine whether gabapentin pretreatment can inhibit CHIMERA-induced axon-glial mechanotransduction in the absence of TRPV4 channels, we injected a single dose of gabapentin into TRPV4^−/−^;Thy1-YFP and TRPV4^+/+^;Thy1-YFP (as WT control) mice 1.5 h before head impact (Fig. 7A). The mice were perfused and fixed either immediately (0 h) or 24 h after head impact. In sharp contrast to GSK219 and GSk279, gabapentin significantly inhibited axonal varicosity induction at both time points in TRPV4^−/−^ mice, as well as in WT mice (Fig. 7B, C). Twenty-four hours (24 h) after CHIMERA, gabapentin pretreatment markedly reduced microglial activation and cortical demyelination in both TRPV4^−/−^ and WT brains (Fig. 7D-G). Interestingly, gabapentin appeared to have less protective effects for axonal varicosity induction and cortical demyelination at 24 h in WT mice than those in TRPV4^−/−^ mice (Fig. 7C, G). These results are consistent with our hypothesis that upregulated Cav2.1 (and Cav2.3) channels in the absence of TRPV4 may mediate axon mechanosensation. However, besides Cav2.1 channel regulation, gabapentin and α_2_δ can regulate many other Cav channels and even non-Cav channel targets [71]. Whether Cav2.1/Ca2.3 alone or with other proteins mediates the genetic compensation remains to be further investigated. Nonetheless, our results show that gabapentin inhibits axon-glial mechanosensation in TRPV4^−/−^ mice, supporting the homeostasis of CNS mechanosensation and mechanotransduction.

## Discussion

Our findings show for the first time that the sequential changes of neuronal and glial cells induced by a concussive head impact include TRPV4-dependent axonal varicosity formation (0 h), followed by excitotoxicity-mediated microglial activation (4 h) and cortical demyelination (24 h). Axonal varicosity induction is most likely accompanied by glutamate release, consistent with the vesicular release of glutamate in axons of white matter or from non-synaptic sites [74]. Our findings indicate that NMDA receptor activation plays a major role in both microglial activation and cortical demyelination, while activated microglia may, in turn, further damage axons and myelin.

Importantly, our results at different time points after head impact indicate that the axon-glial changes and behavioral disturbances can gain substantial recovery to different degrees. The levels of axonal varicosities and microglial activation at 3d but not cortical demyelination significantly reduced compared to their peak levels at 24 h after CHIMERA (Figs. 1 and 2). This is consistent with the recovery of induced axonal varicosities in vitro [28]. Using time-lapse imaging, we found that fluid mechanical stress rapidly (< 5 s) and reversibly (~ 25 min half recovery) induced axonal varicosities in cultured central neurons [28]. After CHIMERA, the axon-glial changes did not completely recover to the sham level two months later. The timeline of axonal varicosity formation appears to correlate with that of behavioral disturbances. This is consistent with the notion that axonal varicosities can deter action potential propagation and hence directly impair neurological functions [75]. Four types of CHIMERA-induced behavioral alterations (in EPM, rotarod, balance beam, and open field) but not recognition memory impairment (in NOR) significantly recovered 1 month after CHIMERA (Fig. 3 and Fig. S4), consistent with the fact that most mTBIs (~ 85%) recover [4, 5]. Our NOR results did show that 0.9 J CHIMERA induced a long-lasting deficit in recognition memory (Fig. S4), which is consistent with the results from other closed-skull mTBI models also showing long-lasting memory deficits [76, 77]. It is important to note that all of our behavioral assays require the mouse to be able to move. Within the first several days after CHIMERA, the movement of impacted mice was markedly impaired. Afterward, the rates of recovery among mice within the same experimental group appeared to vary, leading to large error bars at some time points. Our studies focus on the initial stage of mTBI and do not exclude other factors potentially involved at the later stages. Moreover, some axonal varicosities may continue to form after head impact, which is implicated by the sustained or increased levels of axonal varicosities between 0 h and 24 h after CHIMERA across different brain regions (Fig. 1B and Fig. S1C). Axonal varicosities that developed hours after head impact may result from the degeneration of axonal segments distal to the sites of irreversible initial injury. The mechanisms underlying the slow formation of axonal varicosities in vivo and the formation of residual large varicosities that are sparsely distributed 60d after CHIMERA remain to be investigated in future studies. Taken together, our findings show that a concussive head impact causes sequential axon-glial changes at the cellular level, and these changes gain significant but incomplete recovery after two months. Furthermore, mTBI-induced axon-glial changes can be exacerbated by additional genetic and/or environmental factors, giving rise to long-lasting clinical deficits.

Blocking TRPV4 channel activity with GSK219 or GSK279 offers stronger protection than memantine in CHIMERA by preventing both axonal injury and subsequent glial changes (Figs. 2, 3 and 6). Importantly, GSK219 posttreatment also accelerated the recovery of mTBI and/or prevented secondary injury (Fig. 3K-M and Fig. S5). Since there was no microglial activation in the mouse model for high-frequency subconcussive head impact, memantine was postulated to inhibit NMDA receptors in neurons to block synapse adaptation [37]. In a more severe model of repetitive mTBI, memantine treatment right after the injury inhibited microglial activation and normalized NR2B, LTP, APP, and p-tau [38]. On the other hand, removing microglia before head impact in a severe TBI model did not alleviate cognitive deficits, whereas repopulating microglia attenuated learning deficits and stimulated neurogenesis [78], suggesting that activated microglia may also contribute to neural repair. Mechanical stress can activate NMDA receptors in the absence of agonists [79]. Potentially CHIMERA-activated NMDA receptors in microglia may directly lead to microglial activation, which is blocked by memantine treatment. However, our AAV results show that deleting neuronal TRPV4 prevented CHIMERA-induced axonal varicosities and adjacent microglial activation (Fig. 5). Therefore, our findings indicate that axonal varicosity formation causes microglial activation mainly through glutamate excitotoxicity in the cortex, while additional factor(s) or DAMP(s) associated with axonal varicosities may be also involved in white mater CC and EC (Fig. 2). These CD68-positive microglia may further damage CNS cells, but the possibility that some of these microglia may contribute to tissue recovery can not be excluded. Moreover, our findings indicate that axonal varicosity induction leads to cortical demyelination via NMDA-receptor-mediated excitotoxicity in CHIMERA. This is consistent with the function of NMDA receptors in oligodendrocytes [80]. Abnormal increases of cytoplasmic Ca^2+^ in myelinating oligodendrocytes can disassemble MBP networks and hence induce myelin vesiculation through aberrant phase transition of MBP molecules from cohesive to soluble and non-adhesive state [33]. It is important to note that memantine has other potential targets besides NMDA receptors and displays subunit selectivity of NMDA receptors [81]. Therefore, different blockers of NMDA receptors need to be tested to confirm their roles in mTBI-induced glial changes in future studies. Although our results suggest that ligand-binding activation but not mechanosensitive activation of NMDA receptors mediates the glial changes, we can not rule out the possibility that both forms of activation happen concurrently. Additional analyses of different subunits of NMDA receptors in memantine’s effects and the two forms of activation will clarify these questions in future studies.

Our early study showed that fluid mechanical stress-activated TRPV4 channels and induced aberrant Ca^2+^ increase in axons, which led to axonal varicosity formation via altering microtubule-based axonal transport [28]. Interestingly, a recent study showed that TRPV4 hyperactivation increased intra-axonal Ca^2+^ and hence disrupted axonal transport of mitochondria [82]. Our mass spectrometry results show that TRPV4 deletion altered the expression of many mitochondria-associated proteins in the mouse brain (Fig. 4E and Fig. S6), supporting the link between TRPV4 and mitochondria regulation. Using Cryo-electron tomography, we recently revealed the presence of mitochondria in most axonal varicosities under physiological conditions [83]. Furthermore, another recent study shows that suppressing acute excessive mitochondrial fission inhibits long-lasting injury in the hippocampus in a mouse TBI model [84]. Therefore, the potential regulation of mitochondria by TRPV4 in mTBI-induced axonal varicosities is a promising downstream signaling pathway to be investigated in future studies.

TRPV4 is unlikely to be the only player involved in CNS mechanosensation in mTBI. This is because the sequential changes of axons and glia, as well as behavioral disturbances, can still be induced by CHIMERA (0.9 J) in TRPV4^−/−^ mice (Fig. 4). TRPV4^−/−^ mice were reported to display impaired osmotic and pain sensation, reduced endothelium-dependent vasodilation, and increased susceptibility to obesity [44, 85–87]. Our behavioral assay results focusing on limited aspects of motor, emotional, and cognitive functions did not reveal a significant difference between TRPV4^−/−^ and WT mice in CHIMERA. To search for additional mTBI-related mechanosensor(s) in the absence of TRPV4, we performed genome-wide mass spectrometry analysis on age- and sex-matched WT and TRPV4^−/−^ brains. Our data revealed more than 200 different proteins upregulated in TRPV4^−/−^ brains, including Cav2.1 (P/Q type) and Cav2.3 (R type) channels (Fig. 4 and Fig. S6). Several types of Cav channels were reported to be regulated by mechanical stress and potentially involved in mTBI [88–90]. Our results show that gabapentin pretreatment inhibited CHIMERA-induced axon-glial changes in TRPV4^−/−^ mice, as well as in WT mice (Fig. 7). Gabapentin appeared less effective than GSK219 and GSK279 in terms of preventing axonal varicosity induction and cortical demyelination 24 h after CHIMERA in WT mice (Figs. 3E, 6E and 7C and G). Gabapentin provided stronger protection in TRPV4^−/−^ mice compared to WT mice (Fig. 7C, F, G). Thus, our findings support that Cav channel upregulation may be the compensatory mechanism in TRPV4^−/−^ brain, providing a potential novel link between mTBI and FHM1 at the levels of genetics, cell biology, and pathophysiology. However, it is important to point out that gabapentin may regulate non-Cav channels as well. This hypothesis remains to be validated by future studies. Nonetheless, our results have shown both TRPV4-dependent and -independent pathways in the homeostasis of CNS mechanosensation and mechanotransduction. Importantly, CNS mechanosensation and mechanotransduction are unlikely designed for mTBI or other pathological processes but rather exist with physiological significance as an essential homeostasis in CNS development, function, and/or plasticity. Therefore, genetic deletion of TRPV4 causes drastic expression changes of many different genes, which lead to the restoration of near-normal levels of CNS mechanosensation and mechanotransduction in TRPV4^−/−^ mice, raising the possibility that mTBI susceptibility or head impact tolerance can be affected by genetic factors. The present study suggests that mutations in TRPV4, Cav2.1, or NMDA receptors may belong to such genetic factors. Consistent with this notion, the gain-of-function mutations in Cav2.1 cause FHM1, and FHM1 patients display increased mTBI susceptibility [7, 8]. Whether the patients carrying gain-of-function missense mutations in TRPV4 and with neuromuscular or skeletal disorders [40–43] also show increased mTBI susceptibility is an interesting topic for future clinical research.

In summary, our findings have provided novel mechanistic insights into TRPV4-mediated sequential mechanotransduction of neuronal and glial cells in mTBI. Since NMDA receptor channels and Cav2 channels play essential roles in synaptic transmission of the CNS, TRPV4 may be a better target for both the prevention and treatment of mTBI and related neurological disorders. Based on our new findings, as well as earlier results from others and ours [3, 28, 37, 91], we propose a unified hypothesis that axon mechanosensation regulated by genetic factors plays a key role in triggering sequential neuron-to-glia mechanotransduction after a subconcussive or concussive head impact.

### Limitations of the study

To visualize induced axonal varicosities and other morphological changes of neurons in mTBI mouse models, we used Thy1-YFP transgenic mice, in which a subset of neurons expresses YFP fluorescence proteins under the Thy1 promoter [3, 28]. It is very difficult, if not impossible, to visualize these axonal varicosities by labeling endogenous axonal proteins, in part due to densely packed and intermingled neuronal processes in vivo. However, this approach could not address whether those unlabeled axons formed varicosities in mTBI. In the present study, we have also used AAV-mediated expression of dTomato under the hSyn promoter and hence showed that axonal varicosities can be induced in different subsets of CNS neurons. Nonetheless, additional analyses will be needed to reveal all axonal changes across different brain regions after mTBI in future studies. In the present study, we focus on TRPV4 and its inhibitors while using memantine/NMDAR and gabapentin/Cav2 channels as comparison, since the two TRPV4 blockers have the most striking protective effects in our studies and have not yet been investigated in mTBI. Thorough analyses of glutamate receptors, Cav2 channels, and their inhibitors are beyond the scope of this study.

Our results showed that there was no significant increase in GFAP expression either immediately or long after CHIMERA (0.9 J) (Fig. S3), consistent with early studies [59, 92]. It is important to note that although there was a transient increase of GFAP expression induced by supra-threshold CHIMERA in the corpus callosum [92], our analysis also included the cortex, EC, and hippocampus. Nonetheless, since GFAP is a cytoskeletal protein, we can not rule out the possibility that the expression of other astrocytic proteins remains unchanged by CHIMERA. For instance, both the TRPV4 channel and NMDA receptors were shown to be expressed in astrocytes [93, 94]. Their potential roles in astrocytes in mTBI remain to be determined in future studies.

Moreover, whereas our behavioral assays in the present study have examined motor activity, coordination, balance, motor skill learning, risk avoidance, recognition, and working memory, additional behavioral assays are still needed to reveal all mTBI-related deficits in future studies. Regarding the translational impact of our present study, the findings are directly relevant to the development of a new prophylactic treatment for mTBI and a new therapeutic treatment for mTBI patients within 3 h after a head impact. Whether this strategy may be effective for treating mTBI patients weeks or months after head impact remains to be determined in future studies.

## Materials and methods

### Mouse line crossing and maintenance

C57BL/6L Thy1-YFP-H transgenic mice with the expression of the yellow fluorescent protein (YFP) in a subset of projection neurons were used in the present and our earlier studies (The Jackson Laboratory Stock # 003782) [3, 28]. TRPV4 global KO (TRPV4^−/−^) and floxed (TRPV4^fl/fl^) mouse lines were kindly provided by Dr. Hongzhen Hu at Icahn School of Medicine at Mount Sinai [95]. TPRV4^−/−^ mice were genotyped using TRPV4 WT Reverse Primer (5’-GGTGAACCAAAGGACACTTGCATAG-3’), TRPV4 Mutant Reserve Primer (5’-CAAGGTGAGATGACAGGAGATC-3’) and TRPV4 Forward primer (5’-TGTTCGGGGTGGTTTGGCCAGGATTAT-3’). TRPV4^fl/fl^ mice were genotyped using TRPV4 Mutant Forward primer (5’-TGCTGCTTCTCAAGTGTTCACGCCT-3’), CSD-Lox Forward Primer (5’-GAGATGGCGCAACGCAATTAATG-3’) and CSD-TRPV4 Reverse Primer (5’-ACACAAGACAACACACAACAACCCC-3’). The PCR-based genotyping strategy was used for maintaining and crossing these mouse lines. All experiments were conducted in accordance with the National Institutes of Health Guide for the Care and Use of Laboratory Animals and were approved by the Ohio State University Institutional Animal Care and Use Committee (IACUC).

### CHIMERA (Closed-Head Impact Model of Engineered Rotational Acceleration)

CHIMERA, an mTBI mouse model, was used as previously described [3, 55, 92]. In this model, mice are anesthetized with 4% Isoflurane in oxygen and placed in a supine position on a foam/plastic cradle, with a 50 g free-floating chrome-coated steel piston under the dorsal surface of the mouse head. The pressure regulator of the accumulator air tank was adjusted to 5.36 psi, resulting in a piston impact on top of the mouse’s head (the midpoint between bregma and lambda) at a speed of approximately 5.8 m/s and with an impact energy of 0.9 joules (0.9 J) [3]. This CHIMERA model mimics the conditions of concussion or mTBI in real-life situations and provides precise control over the impact energy, ensuring that all mice are impacted in the same direction and position with the same impact energy. Before the head impact, mice were administered subcutaneously with meloxicam (1 mg/kg) and saline (1 mL/100 g body weight) for pain control and hydration. After the head impact, the mice were placed in a warm cage and monitored for recovery of right reflexes. Any mice that did not properly recover in their heart beating and breathing, or that exhibited any sign of bleeding, were excluded from the analysis.

### Behavioral assays

Rotarod: The motor function of mice was assessed using the rotarod apparatus (Panlab 76–0770, Barcelona, Spain) as previously described [66]. The rotarod apparatus consisted of a rotating rod, a sensor below the rod, and a digital timer. The testing began by placing each mouse on the rod, which was initially set at 4 rpm for 30 s (sec) to allow for acclimation. The speed gradually increased to 40 rpm over 5 min (min). Mice that were unable to maintain their grip on the rod and fell onto the sensor below were recorded as having a latency to fall. The time that each mouse remained on the rod was recorded for a maximum of 5 min, and the test was performed five times per mouse.

Balance Beam: Motor coordination and vestibular function of mice were assessed using a balance beam test as previously described [66]. The balance beam was 1 m long and 5 mm wide, and elevated 1 m above the desktop. The surface of the beam was smooth. A box with home cage bedding was placed at one end of the beam to attract the mice. Each mouse was placed on the opposite end of the beam and we recorded the time it took to reach the box. If a mouse fell off the beam or failed to reach the box within 60 s, it was assigned a score of 60 s. Each mouse underwent three trials with approximately 10 min of rest between trials.

Elevated plus maze: Mice were put into a plus-shaped maze as described in our previous papers [66, 96], which is 74 cm above the ground, and each arm of the maze is 34 cm long and 5 cm wide. Among the four arms, one pair of arms is an aisle with walls blocking the view, and the other pair of arms is a flat walkway without enclosure. Mice spend most of their time hiding in closed arms, occasionally venturing into open arms. Within 5 min after placing the mice in the maze, a video camera fixed to the ceiling recorded the tracks and total distance traveled by the mice. When the center point of the mouse enters the open arms area, it is considered to be in the open arms. Then EthoVision software was used to count the time of the mice in the open arms and to calculate the proportion of the time spent in the open arms of the mice in the total time of 5 min.

Open field test: The open field test was conducted to evaluate the locomotion and risk avoidance behavior of mice as we previously described [66, 96]. The open field arena was constructed as a cube box with a side length of 40 cm, with an opening on the top side. Upon placement in the arena, mice typically remained near the wall of the arena and occasionally ventured toward the center. A camera positioned directly above the arena recorded the mice’s movements for 10 min. Recorded footage was analyzed using Ethovision software to determine the total distance moved by the mouse, which served as an indicator of locomotion. Additionally, the central area of the arena, measuring 25 cm x 25 cm, was defined as the risk area, and the percentage of time spent by the mouse in the central area during the 10-min trial was used as a measure of risk avoidance behavior.

Novel object recognition test: The memory function of mice was assessed using a novel object recognition test as we previously described [66]. The test consisted of two phases: familiarization and testing. In the familiarization phase, two identical plastic objects were placed symmetrically in a 40 cm × 40 cm open field arena and allowed mice to explore freely for 5 min. In the testing phase, after 4 h (h), one of the objects was replaced with another plastic object of the same material but different in shape and size, and the mice’s exploration behavior was observed for another 5 min. It was considered that mice were exploring an object when their center point was within a radius of 5 cm of the object. The time spent by mice exploring the novel object (Tn) and the familiar object (Tf) was measured, and the discrimination index as (Tn-Tf)/(Tn + Tf) was calculated. A positive discrimination index indicates a preference for the novel object, reflecting normal memory function.

Y-maze test: The spatial working memory of mice was evaluated using a Y-maze spontaneous alternation test as we previously described [66, 96]. The test was conducted in a Y-shaped maze with three arms of equal length (40 cm) and width (8 cm). The arms had opaque plastic walls (20 cm high) to prevent visual cues. We placed each mouse in the center of the maze and allowed it to explore the three arms freely for 5 min. We recorded the number of arm entries and the sequence of arm choices for each mouse. An arm entry was defined as when all four feet of the mouse were within the arm. A triad was a sequence of three consecutive arm entries. A spontaneous alternation was a triad in which the mouse visited all three arms in any order. We calculated the percentage of spontaneous alternation as the ratio of actual to possible alternations multiplied by 100. A higher percentage of spontaneous alternation indicates better spatial working memory.

### Cardiac perfusion and brain fixation

Cardiac perfusion and brain fixation are performed as we described in our previous publications [3, 83, 97]. In brief, anesthesia was induced through intraperitoneal injection of 10 mg/ml Euthasol at 0.5 ml per mouse, and it was ensured that the mouse was unresponsive to tail/toe pinches before proceeding. The mouse was then secured in the supine position, and an incision was made through the brain to expose the thoracic field. The beating heart was secured with a hemostat, and a needle was immediately inserted into the left ventricle. Next, the right atrium was cut with scissors, and 1x PBS was infused until the fluid exiting the right atrium was entirely clear. The perfusion fluid was then switched to 4% formaldehyde in PBS. After decapitating the mouse and removing the brain, we cut the cranium along the mid-sagittal suture and gently removed the brain using forceps. The brain was cut coronally into three pieces on a glass matrix block and soaked in the fixative for 1 h at 4 °C. After removing the fixative, we soaked the brain pieces in 30% sucrose in PBS and kept them at 4 °C until they sank to the bottom of the sucrose tube. Finally, we embedded the brain pieces in Tissue-Tek O.C.T. Compound with a foil cube mold and kept them at -80 °C. The tissue blocks were sectioned into 40 μm slices using a cryostat microtome for subsequent analyses.

### Chemicals and antibodies

Antibodies used in this study include a rabbit polyclonal anti-degraded MBP antibody that recognizes damaged MBP proteins (1:2000, anti-dMBP; Catalog #: AB5864; Chemicon), a rat monoclonal anti-MBP antibody (1:200; Catalog #: ab7349; abcam), a rabbit polyclonal anti-TRPV4 antibody (1:200; Catalog #: LS-A8583; LifeSpan BioSciences, Seattle, Washington, USA), rabbit polyclonal anti-Cav2.1, anti-Cav2.2 and anti-Cav2.3 antibodies (Catalog #s: ACC-001, ACC-002 and ACC-006; Alomone Labs, Jerusalem, Israel), a goat polyclonal anti-GFAP antibody (1:200; Catolog #: Ab53554; Abcam Inc. Waltham, MA, USA), and a rat monoclonal anti-CD68 antibody (1:200; Catalog #: MCA1957GA; Bio-Rad; Hercules, CA, USA), as well as Cy3 and Cy5-conjugated secondary antibodies (Jackson ImmunoResearch Laboratories, West Grove, PA, USA). The nuclear dye Hoechst was purchased from Vector Laboratories (Catalog #: FL-1351–2; Burlingame, CA, USA).

### Immunofluorescence staining

The immunofluorescence staining protocol was adopted from our previous publications [3, 98, 99]. Each brain slide was incubated in PBS/1% Triton X-100 for 1 h at room temperature (RT), then washed and blocked with 2.5% normal goat or donkey serum in washing buffer (PBS/0.02% Triton X-100) for another hour at RT. The origin of the blocking serum matches the secondary antibody host. Primary antibodies were diluted in blocking solution (1:100 or 1:200, depending on the antibody) and applied to each slide, covering each brain slide with parafilm. The slides were incubated overnight at 4 °C, then rinsed with washing buffer 7 times for 5 min each at RT. Secondary antibodies were also diluted in blocking solution (1:100 or 1:200, depending on the antibody) and added to each slide, followed by aluminum foil to protect from light. The slides were incubated for 3 h at RT, then washed once with washing buffer. Hoechst was added to the washing buffer (1:10000) and applied to each slide for 10 min at RT, then rinsed 7 times for 5 min each at RT. Finally, Tris-buffered Fluoro-Gel mounting media Electron Microscopy Sciences, Hatfield, PA, USA) was added, and the slides were cover-slipped with Fisherbrand Microscope Cover Glass.

### Western blotting

The procedure for the Western blotting analysis of ion channel proteins in the mouse brain was previously described [28, 66, 100]. In brief, the brain tissue was first homogenized and solubilized in a buffer containing 50 mM Tris-Cl, pH 7.4, 150 mM NaCl, 1% Triton X-100, and complete protease inhibitors at 4 °C for 2 h. The resulting mixture was then centrifuged at 10,350 rpm for 30 min at 4 °C to remove insolubilized materials. The supernatant was resolved in SDS-PAGE electrophoresis, transferred onto a PVDF membrane, and blotted with a specific antibody, followed by an HRP-conjugated secondary antibody. An ECL kit was used to detect protein bands. To ensure an approximately equal amount of proteins were loaded in WT and KO mouse lanes, we used a Colloidal Blue staining kit (Cat. #LC6025, Invitrogen, Massachusetts, USA) to reveal all protein bands [66], as well as anti-β-tubulin (Millipore) Western blot, to fine calibrate the loading volume for each lane.

### Fluorescence microscopy and image capture

For low-magnification image capture below 20×, a Zeiss Axiophot (Carl Zeiss AG, Oberkochen, Germany) upright microscope was utilized with objectives 4×, 10×, and 20× Plan Apo. Grayscale images were captured at the wavelength corresponding to the coupled fluorescence and Hoechst nuclear dye. These grayscale images were merged into pseudo-color images using ImageJ and/or Photoshop. Quantification was performed using low-magnification raw images with the same exposure time, as previously described [3, 66, 83].

### Confocal microscopy and 3D reconstruction

For high-magnification image acquisition, a Zeiss LSM 900 confocal system from Airyscan SR and an Andor Revolution WD spinning disk laser confocal system with Neo sCMOS camera were used with 63x and 100x immersion oil objectives, respectively. Grayscale images at wavelengths corresponding to different secondary antibody-coupled fluorophores and Hoechst stains were captured using 405 nm, 488 nm, 515 nm, 561 nm, or 640 nm laser lines. Fiji ImageJ was used to merge images from different wavelengths at the same location to form colored images. Additionally, Z-stack images for selected regions of interest with a step size of ~ 0.20 μm were captured, and 3D videos were generated using Fiji ImageJ.

### Drug treatment

GSK2193874 (GSK219) (Catalog #: 5106; Tocris Bioscience, Bristol, UK) was administered to mice via gavage at a dosage of 20 mg/kg body weight. For the pretreatment experiment, mice were gavaged with one dose of GSK219 3 h before the head impact in CHIMERA (0.9 J). Then, the pretreated mice are analyzed using anatomical or behavioral assays at different time points after the impact. In the posttreatment experiments, mice were gavaged with one dose of GSK219 3 h after the head impact and analyzed at different time points. In the memantine treatment experiment, we administered memantine hydrochloride (Sigma Aldrich, M9292, St Louis, MO) via intraperitoneal injection with a dosage of 10 mg/kg body weight, 1.5 h before and 3 h after the impact. In the GSK2798745 (GSK279)(Catalog #: HY-19765; MedChemExpress LLC, Monmouth Junction, NJ, USA) [67] treatment experiment, we administered GSK279 via tail vein injection with a dosage of 20 µg/kg mouse body weight 1 h before the impact. In the gabapentin treatment experiment, we administered gabapentin (Catalog #: PHR1049; MilliporeSigma, Burlington, MA, USA) via IP injection (50 mg/kg mouse body weight) 1.5 h before head impact. Some mice were perfused immediately after the impact (0 h), while the rest of the mice were perfused 24 h after the impact (24 h). The timing of the drug pretreatment was based on each drug’s established pharmacokinetics and delivery route to ensure it reached the optimal level in the brain at the time of head impact.

### Mass spectrometry and bioinformatics analysis

Mass spectrum analysis of both WT and TRPV4^−/−^ brain tissues was conducted at the Campus Chemical Instrument Center Mass Spectrometry and Proteomics Facility at The Ohio State University. After the mice were euthanized, brain tissues were immediately frozen in liquid nitrogen and kept at -80 °C. Protein extraction was performed by adding 3 mg of brain tissue to a test tube and digesting it in a solution containing RapiGest or ProteaseMax. The supernatant was then transferred to a new LoBind tube, and the protein concentration was measured using a Thermo Fisher Qubit Fluorometer. Alkylation of 30 µg of extracted proteins was performed with dithiothreitol (DTT) and iodoacetamide (IAA) and reconstituted with trypsin (Promega). Brain samples were analyzed on a Thermo Scientific Q Exactive Plus High Resolution Accurate Mass (HRAM) mass spectrometer with a C18 Easy-Spray column (Thermo Scientific). The mass spectrometer acquired full-scan mass spectra from m/z 375 to 1575 at a resolution of 70,000. The top 15 most abundant precursor ions per full MS1 spectrum were selected for fragmentation, and an isolation window of 1.6 m/z was used for fragmentation with a normalized collision energy of 30. Tandem mass spectra (MS2) were acquired at a resolution of 17,500. Peptide and protein identifications were obtained using Thermo Proteome Discoverer software and the Sequest search algorithm against the UniProt human database. Gene Ontology term enrichment is conducted using GOnet [101].

### AAV vector and stereotaxic microinjection

AAV9 particles prepared from pAAV-hSyn-Cre-P2A-dTomato (107738-AAV9, Addgene) were injected into the TRPV4^fl/fl^ (or WT as control) mouse brain with the manufacturer’s original Titer (≥ 7 × 10¹² vg/mL) on a stereotaxic apparatus as previously described [66, 99]. This virus targets and infects neurons in the mouse brain. The Cre-Lox system allowed for the conditional knockout of TRPV4 in the infected neurons. The day before the operation, the mice were fed with 30 mg/kg/day ibuprofen in drinking water and continued for 3–7 days after the operation. Before surgery, the mice were weighed, and ophthalmic lubricant was applied to their eyes to prevent dryness. The mice were anesthetized with a mixture of ketamine (50–100 mg/kg) and xylazine (5–20 mg/kg), and received a first dose of buprenorphine (0.05–0.1 mg/kg). After shaving the mouse’s head and aseptically preparing the brain, the mouse was mounted on a stereotaxic apparatus. A midline incision (approximately 1 cm) was made in the scalp to expose the skull. The bregma and lambda points were identified, and the left and right sides of the mouse were leveled using a stereotaxic measurement. Using the bregma as a reference point, the target injection point (AP: -2.2 mm; ML: -1.0 mm; DV: -1.0 mm) was determined directly above the skull, and a 1-mm-diameter hole was drilled in the skull at this point. A guide cannula was then implanted through the burr hole into the selected brain region to determine the depth of the injection point. pAAV-hSyn-Cre-P2A-dTomato (~ 1–10 µl) was injected slowly at 0.25 µL/min using a syringe connected to the guide cannula. The cannula was left in place for 2 min after the injection was completed, and the cannula was then gradually and slowly withdrawn. After the surgery, the mice were allowed to recover for 4 weeks. The success of AAV infection in the injected brain area was verified using dTomato fluorescence.

### Transmission electron microscopy (TEM)

To examine the ultrastructure of the mouse brain, transmission electron microscopy (TEM) was used. Cardiac perfusion with fixative (2% paraformaldehyde and 2.5% glutaraldehyde in 0.1 M phosphate buffer) was performed to ensure proper fixation. The brain tissue was then removed and fixed in 4% glutaraldehyde for at least 24 h. The gray and white matter samples were prepared at the Campus Microscopy and Imaging Facility at The Ohio State University. After fixation, the brain tissue was post-fixed in 1% osmium tetroxide (Ted Pella, Inc., Redding, CA, USA) and then en bloc stained with 1% uranyl acetate (Ted Pella Inc.). A series of graded ethanol and then 100% acetone were used to fully dehydrate the brain tissue. The fully dehydrated brain tissue was embedded in Eponate 12 epoxy resin (Ted Pella Inc.) to make resin blocks, which were then cut with a Leica EM UC6 ultramicrotome (Leica Microsystems, Inc., Deerfield, IL, USA) into thin sections of about 80 nm thickness. The thin sections were examined with a FEI Tecnai G2 Spirit BioTwin TEM (Thermo Fisher Scientific, Waltham, MA, USA) operating at 80 kV at the Campus Microscopy and Imaging Facility. Images were captured using a Macrofire digital camera (Optronics, Inc., Chelmsford, MA, USA) and AMT image capture software (Advanced Microscopy Techniques, Woburn, MA, USA).

### Statistical analysis

Sample sizes were chosen based on our previous experiments and publications, as well as related literature in the field. Mice of matching sex and age were allocated into various groups. Since no sex difference was detected in our experiments, the results from male and female mice were combined. In each experimental group, approximately 50% of the mice were male and the other 50% were female. No formal randomization was conducted. Investigators were blinded to genotype and treatment groups during behavioral analysis. In AAV injection experiments, post hoc analyses of viral infection and protein expression were used to exclude mice with no or incorrect AAV infection from behavioral results. Statistical analysis was conducted using SigmaPlot 15.0 software. To assess normality, the Shapiro-Wilk test was employed, and the homogeneity of variances was evaluated using the Brown-Forsythe test. Results were presented as the mean ± SEM. Two-tailed Student’s t-test was used for comparisons between two groups. One-way ANOVA followed by Dunnett’s test was used for comparing two or more groups to one control group. (*) *p* < 0.05, (**) *p* < 0.01, and (***) *p* < 0.001 were considered statistically significant.

## Supplementary Information

Below is the link to the electronic supplementary material.

Supplementary Material 1

## Data Availability

All data are available in the main text or the supplementary materials.
