# Supplementary material for: Blocking axon-glial mechanotransduction to prevent concussive brain injury
Source: Acta Neuropathol Commun. 2025 Sep 29;13:205. doi: 10.1186/s40478-025-02117-6 (PMC12481915; doi:10.1186/s40478-025-02117-6)
Supplement: Supplementary file 1 — Supplementary Material 1 [file 40478_2025_2117_MOESM1_ESM.docx]

**Supplementary Materials for**

**Blocking Axon-Glial Mechanotransduction to Prevent Concussive Brain Injury**

Chao Sun^1,3^, Di Ma^2,3^, Jacob Hansen^2.3^, Jeffrey R. Tonniges^4^, Hongzhen Hu^5^, Liwen Zhang^6^, and Chen Gu^1-3^*

**This PDF file includes:**

Supplementary Figs. S1 to S9


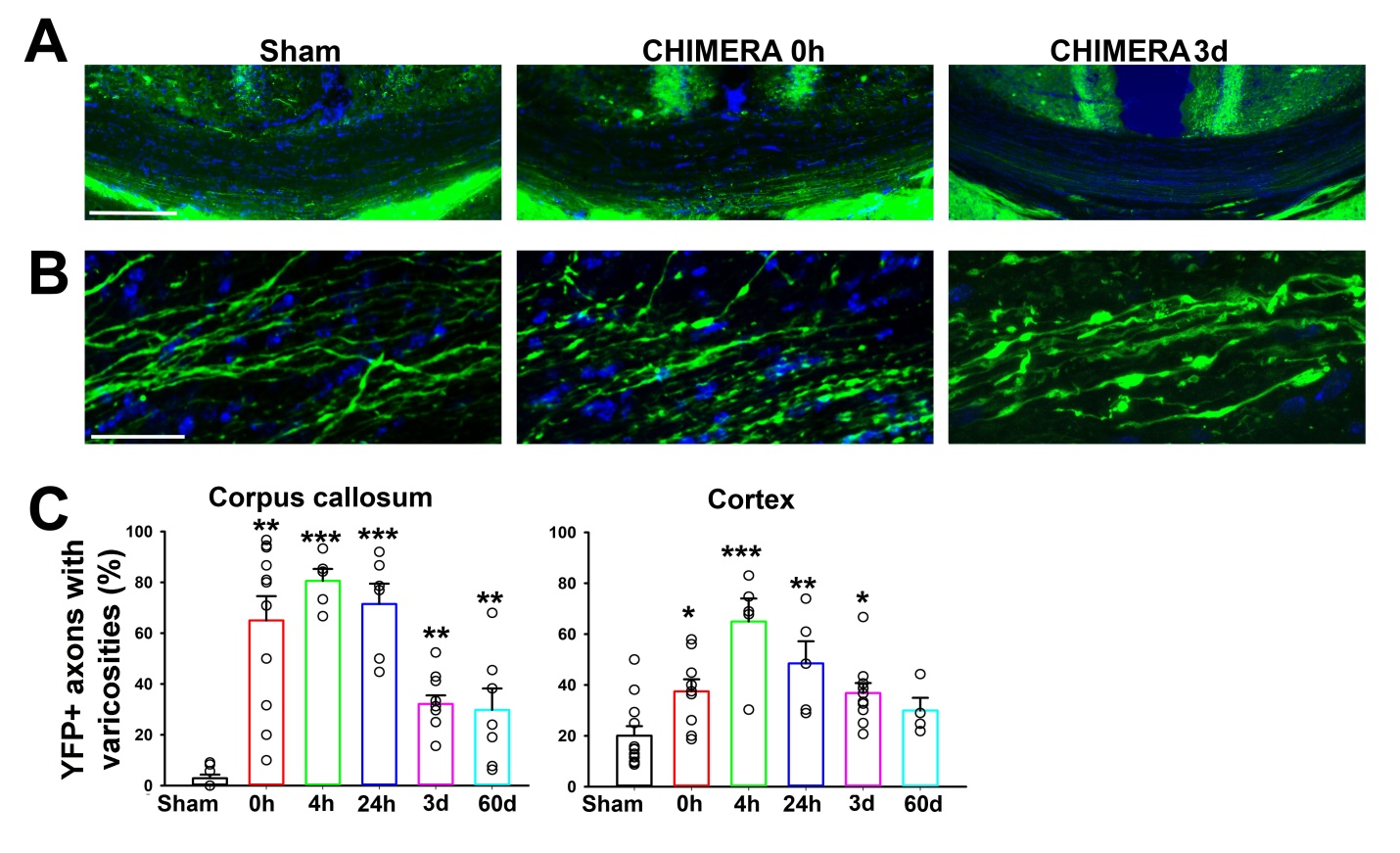


**Supplementary Fig. S1. CHIMERA-induced axonal varicosities in different brain regions are partially reversible.**

Images from the corpus callosum (CC) (**A**) or the external capsule (EC) (**B**) of Thy1-YFP transgenic mice that received no impact (Sham, left), or 0h (middle) and 3d (right) after one 0.9J head impact in CHIMERA. YFP signals are in green and Hoechst in blue.

(**C**) Summary of percentage of YFP+ axons with varicosities in the CC (left) or the cortex (right) at different time points after CHIMERA. Mouse numbers: 6 (Sham), 6 (0h), 3 (4h), 4 (24h), 5 (3d), and 3 (60d). 1-3 images from each mouse are included for each brain region. One-way ANOVA followed by Dunnett’s test: * *p* < 0.05, ** *p* < 0.01, *** *p* < 0.001.

Scale bars, 250 μm in (**A**), and 40 μm in (**B**).

**
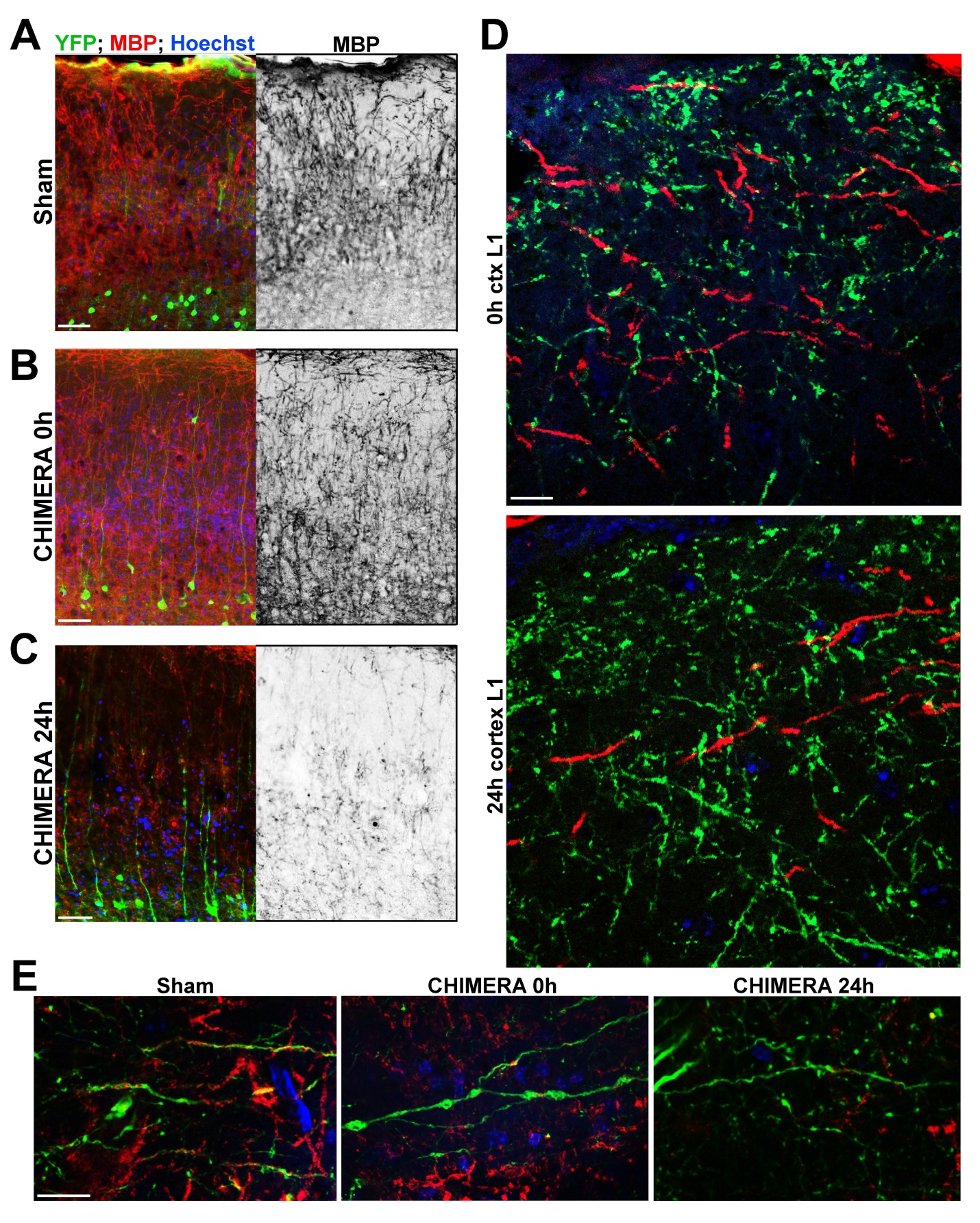
**

**Supplementary Fig. S2. Delayed cortical demyelination induced by CHIMERA.**

Low-mag images of the cortex of the Thy1-YFP transgenic mice received no impact (Sham) (**A**), 0h (**B**), and 24h (**C**) after CHIMERA (0.9J). The anti-MBP staining signals are in red in merged images (left) and inverted in grayscale images (right). YFP is in green and Hoechst is in blue.

(**D**) Confocal images of cortical layer 1 at 0h (upper) and 24h (bottom) after CHIMERA. MBP, red; YFP, green; Hoechst, blue.

(**E**) Confocal images of cortical layer 6 from Sham mice (left), or 0h (middle) and 24h (right) after CHIMERA.

Scale bars, 250 μm in (**A**)-(**C**), 30 μm in (**D**) and (**E**).

**
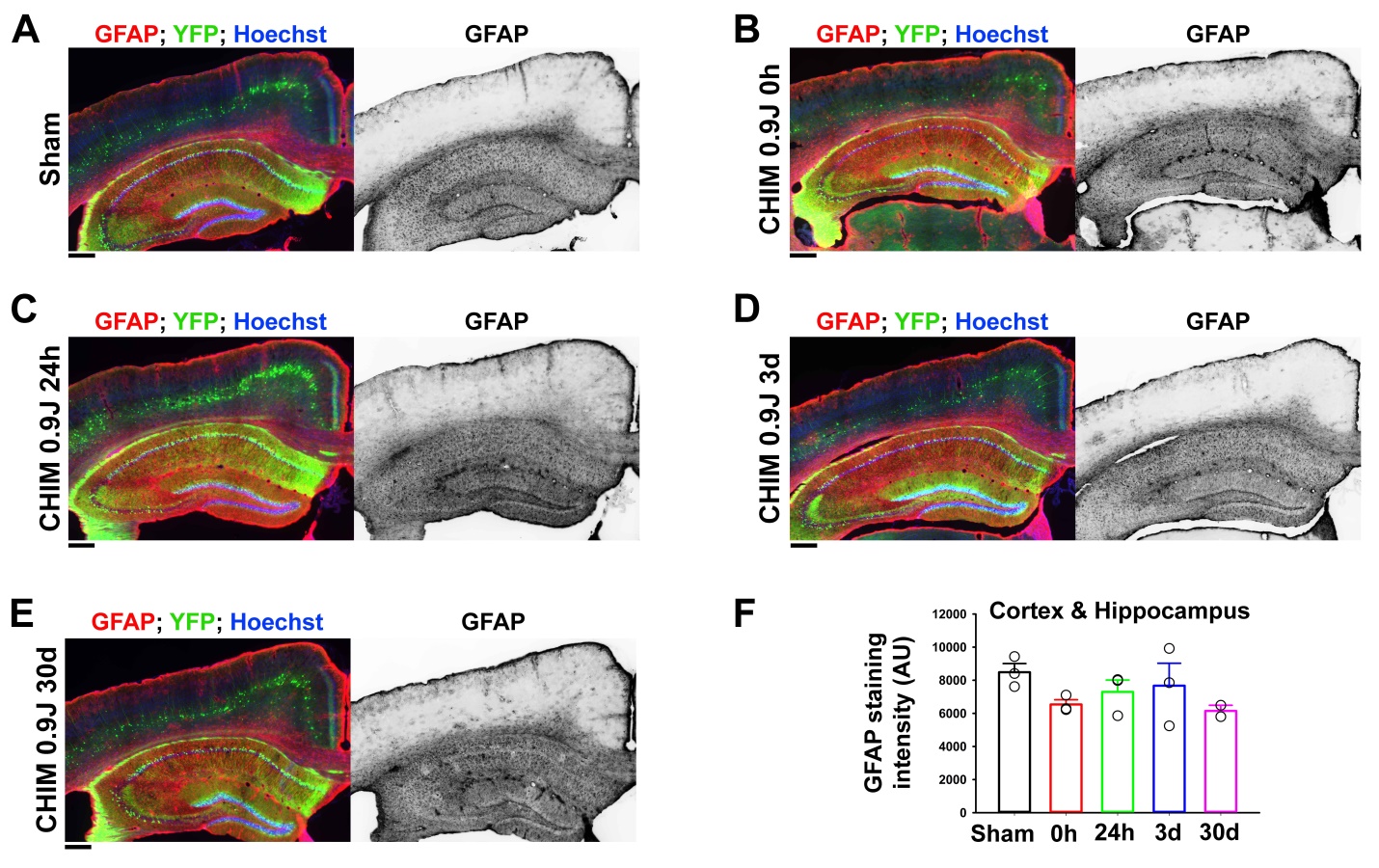
**

**Supplementary Fig. S3. No increase of GFAP expression by CHIMERA.**

Coronal sections (including the cortex and hippocampus) of the Thy1-YFP transgenic mice received no impact (Sham) (**A**), or 0h (**B**), 24h (**C**), 3d (**D**), and 30d (**E**) after CHIMERA (0.9J). The anti-GFAP staining signals are in red in merged images (left) and inverted in grayscale images (right). YFP is in green and Hoechst is in blue.

(**F**) Summary of GFAP staining intensities in the cortex and hippocampus. One-way ANOVA followed by Dunnett’s test: no significant difference. Mouse number n=3 in all conditions.

Scale bars, 300 μm.

**
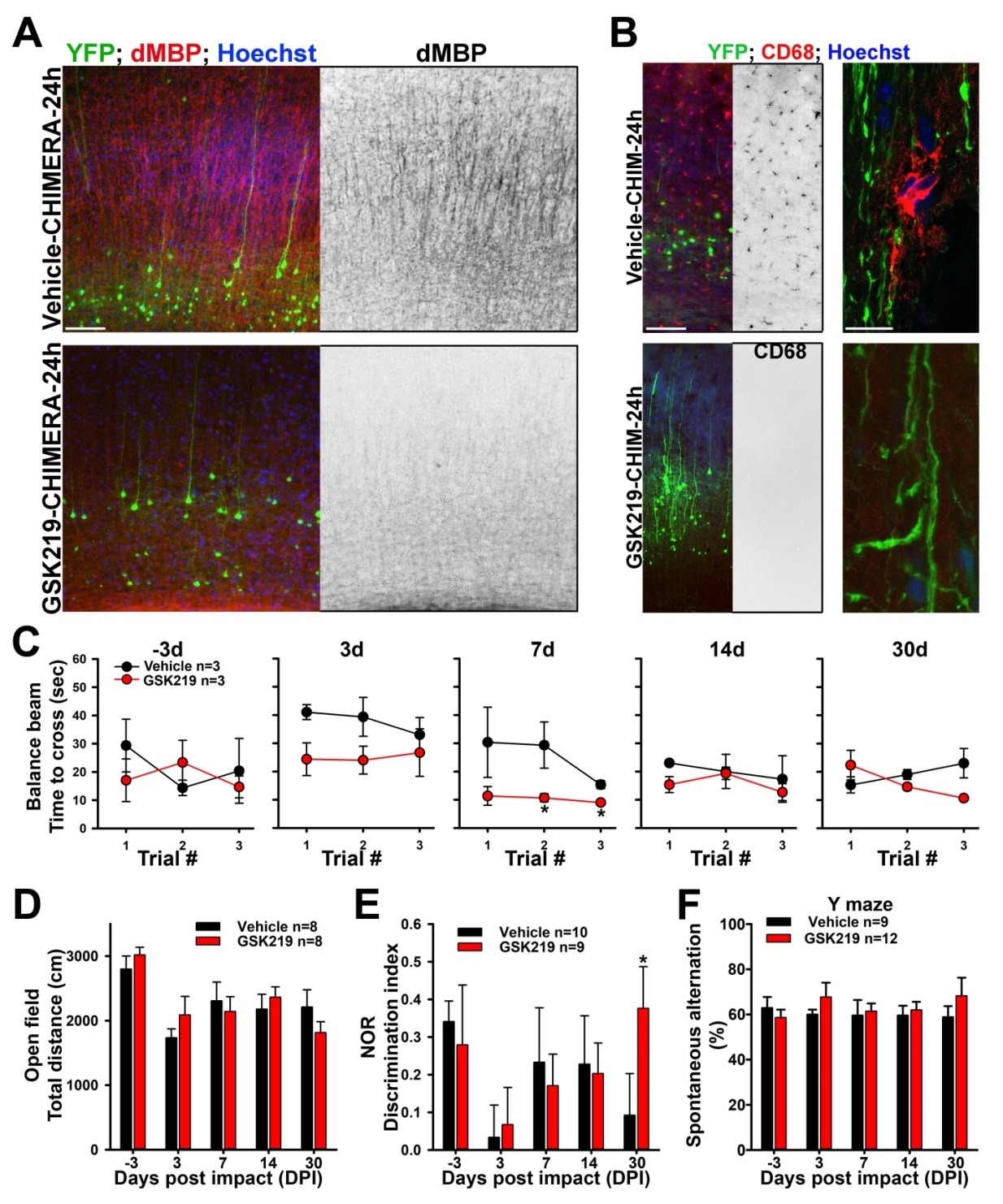
**

**Supplementary Fig. S4. GSK219 pretreatment markedly reduced cortical demyelination, microglial activation, and behavioral alterations caused by CHIMERA.**

(**A**) GSK219 pretreatment markedly reduced cortical demyelination 24h after CHIMERA. The dMBP staining signals are in red in merged images (left) and inverted in grayscale images (right), YFP in green, and Hoechst in blue.

(**B**) GSK219 pretreatment eliminated CD68 signals in the cortex and EC 24h after CHIMERA. The CD68 signals are in red in merged images and inverted in grayscale images (middle). Confocal images are on the right.

(**C**) Balance-beam results before CHIMERA (-3d), and 3d, 7d, 14d, and 30d after CHIMERA with vehicle or GSK219 pretreatment. Unpaired t-test: 7d trial #2 **p* = 0.0444; 7d trial #3 **p* = 0.0121.

(**D**) The total travel distance of mice in CHIMERA with vehicle or GSK219 pretreatment.

(**E**) Summary of discrimination index ((T_N_-T_F_)/(T_N_+T_F_) in the novel-object-recognition (NOR) test at different time points in CHIMERA with vehicle or GSK219 pretreatment. Unpaired t-test: 30d **p* = 0.0164.

(**F**) Summary of spontaneous alternation (%) in the Y-maze test at different time points in CHIMERA with vehicle or GSK219 pretreatment.

Unpaired t-test: *, *p* < 0.05. Scale bars, 250 μm in (**A**) and (**B**) left; 30 μm in (**B**) right.

**
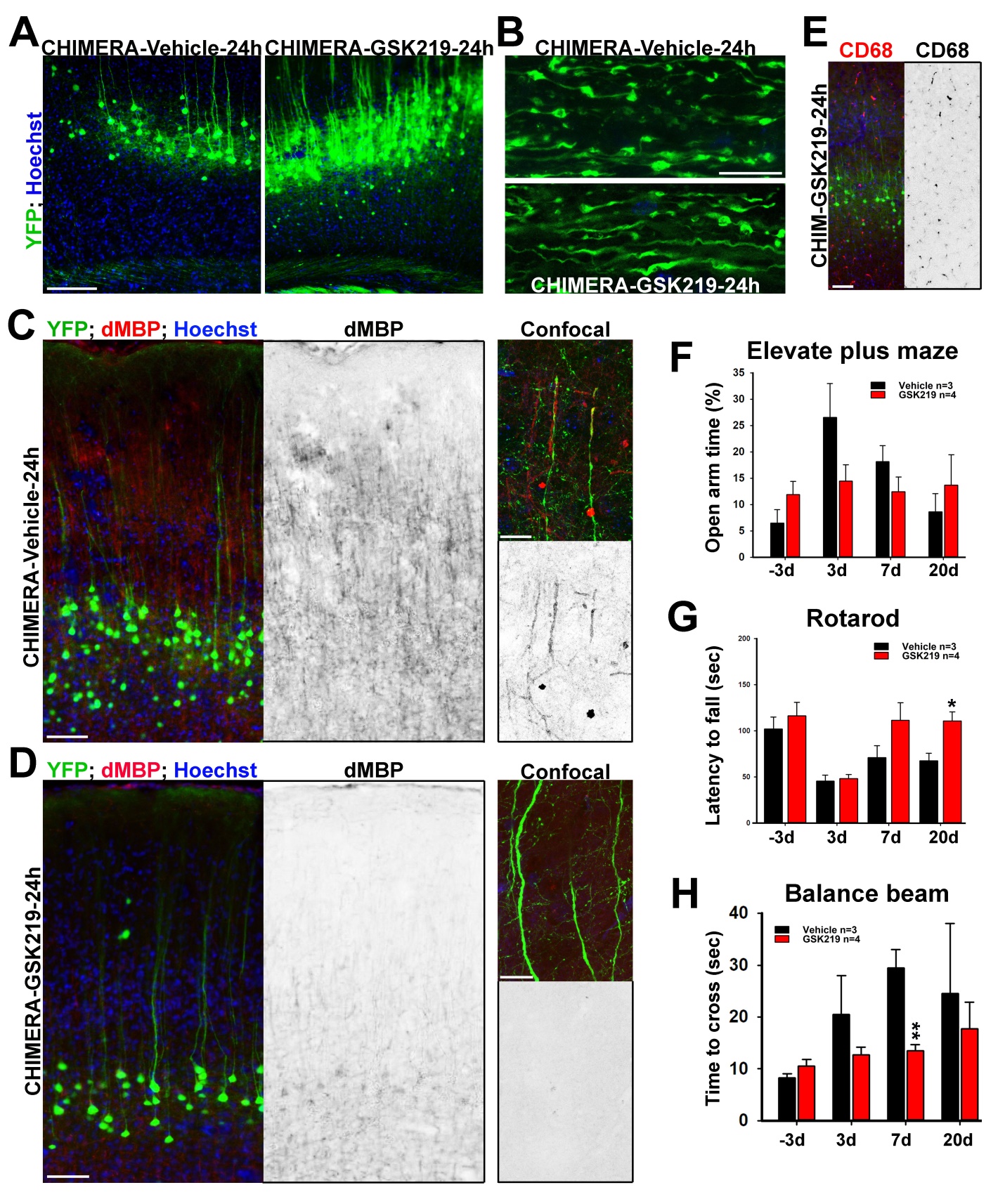
**

**Supplementary Fig. S5. GSK219 post-treatment reduced axonal varicosity level and cortical demyelination but not microglial activation 24h after CHIMERA.**

Low magnification (**A**) and confocal (**B**) images of the cortex and EC of Thy1-YFP transgenic mice 24h after CHIMERA with vehicle or GSK219 post-treatment.

(**C**) Low magnification (left) and confocal (right) images of the mouse cortex 24h after CHIMERA with vehicle post-treatment. The dMBP staining signals are in red in merged images and inverted in gray-scale images.

(**D**) Images 24h after CHIMERA with GSK219 post-treatment.

(**E**) GSK219 post-treatment did not inhibit microglial activation revealed by increased CD68 staining signals. The CD68 staining signals are in red in the merged image and inverted in the gray-scale image.

Scale bars, 200 μm in (**A**), (**C**) left, (**D**) left, and (**E**); 20 μm in (**B**), (**C**) right, and (**D**) right.

(**F**) The effect of GSK219 post-treatment on the EPM result, the percentage of open-arm time, in CHIMERA. Mouse numbers are provided in the chart.

(**G**) The effect of GSK219 post-treatment on the rotarod test (the 5^th^ and last trial), Latency to fall, in CHIMERA. Unpaired t-test: 20d *p* = 0.0481.

(**H**) The effect of GSK219 post-treatment on the balance-beam test (the 3^rd^ and last trial), Time to cross, in CHIMERA. Unpaired t-test: 7d *p* = 0.00458.

Unpaired t-test: *, *p* < 0.05; **, *p* < 0.01.

**
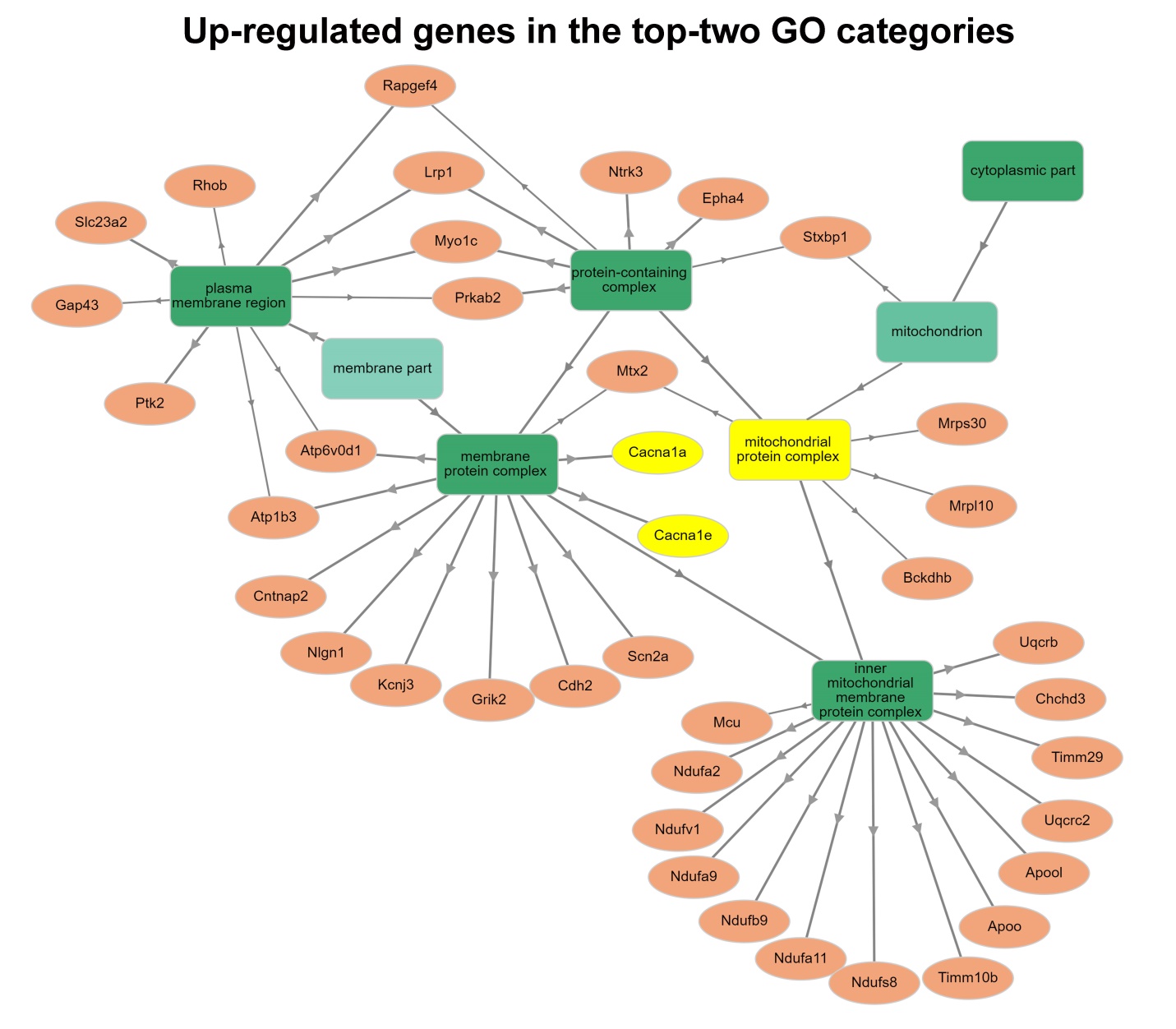
**

**Supplementary Fig. S6. Upregulated proteins in the membrane protein complex and mitochondrial protein complex.**

Upregulated proteins in the top two GO categories are the membrane protein complex and mitochondrial protein complex. Individual proteins are shown in pink ovals and GO categories are in green rectangles. Cacna1a (Cav2.1) and Cacna1e (Cav2.3) proteins and mitochondrial protein complexes are highlighted in yellow.

**
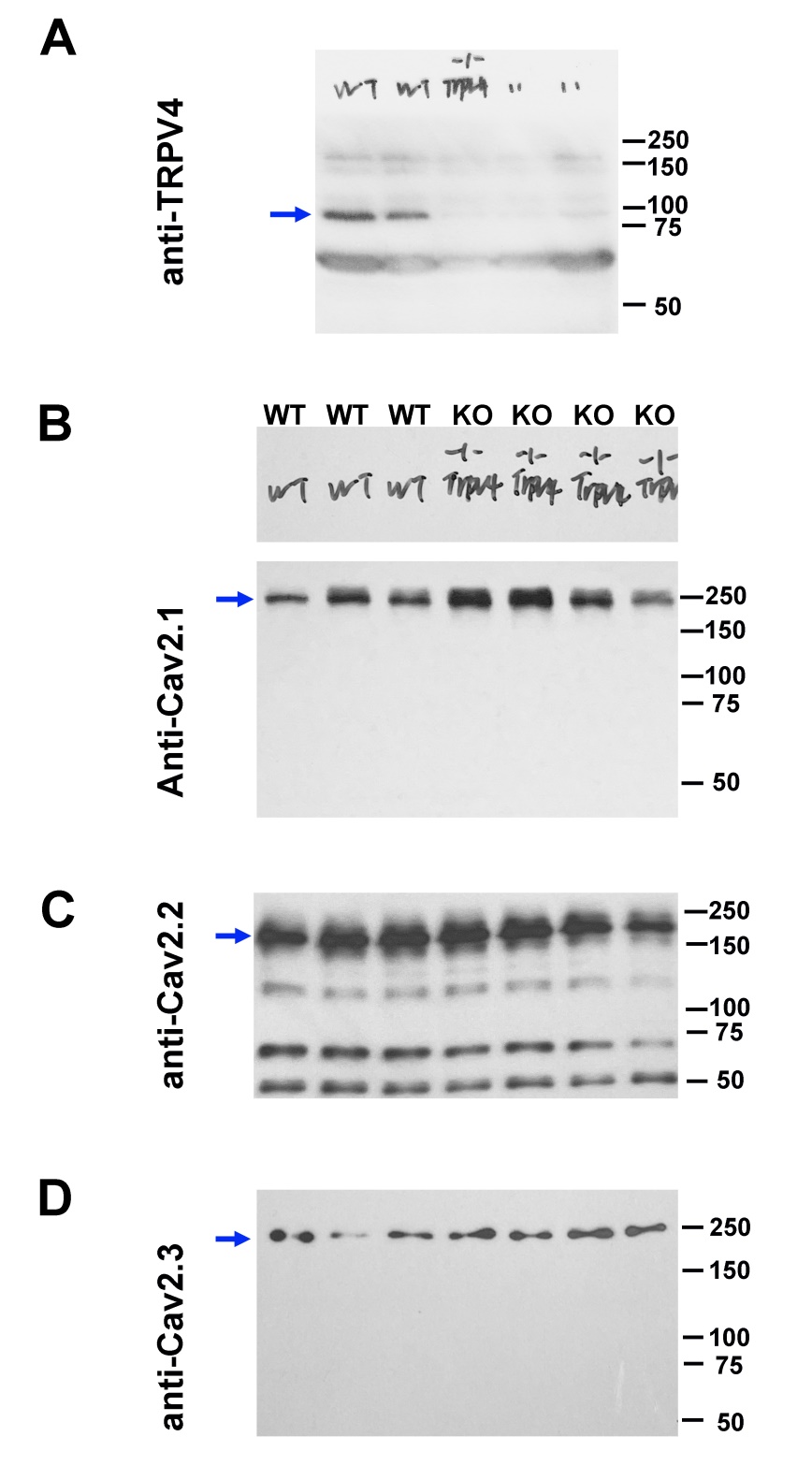
**

**Supplementary Fig. S7. Western blots included in Figure 4.**

Brain lysates from wild type (WT) or TRPV4^-/-^ (KO) mice were resolved in SDS PAGE and blotted with an anti-TRPV4 (**A**), an anti-Cav2.1 (**B**), an anti-Cav2.2 (**C**), or an anti-Cav2.3 (**D**) antibody. Numbers on the right, molecular weights in kDa. Blue arrows on the left, the full-length protein bands.

**
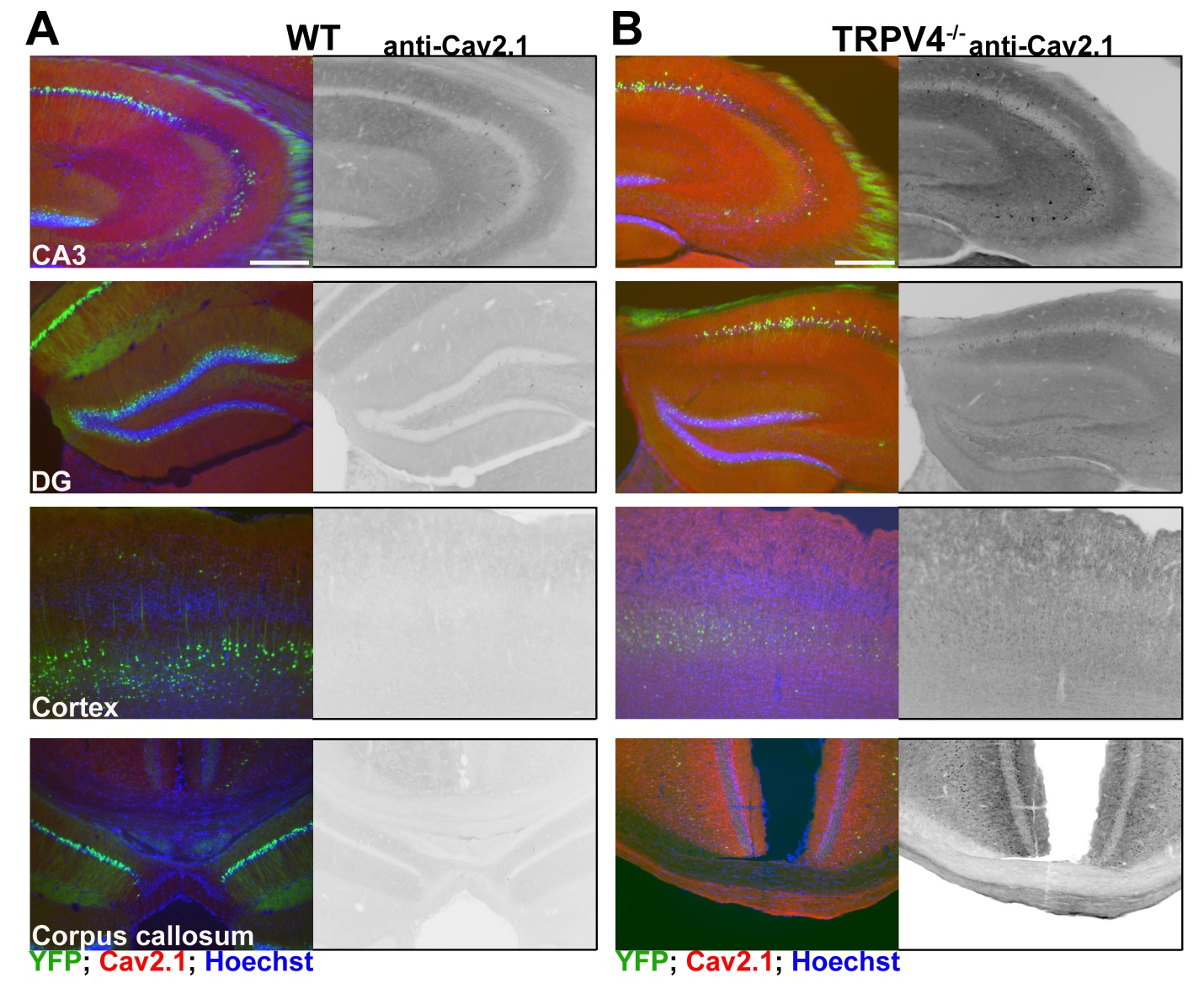
**

**Supplementary Fig. S8. Upregulation of Cav2.1 proteins in the brain of TRPV4^-/-^ mice revealed by immunostaining.**

(**A**) Anti-Cav2.1 staining signals in hippocampal CA3 and dentate gyrus, the cortex, and the corpus callosum of WT (TRPV4^+/+^;Thy1-YFP) mice.

(**B**) Anti-Cav2.1 staining signals in hippocampal CA3 and dentate gyrus, the cortex, and the corpus callosum of TRPV4^-/-^ (TRPV4^-/-^;Thy1-YFP) mice. The anti-Cav2.1 staining signals are in red in merged images (left) and inverted in gray-scale images (right).

Scale bars, 400 μm.

**
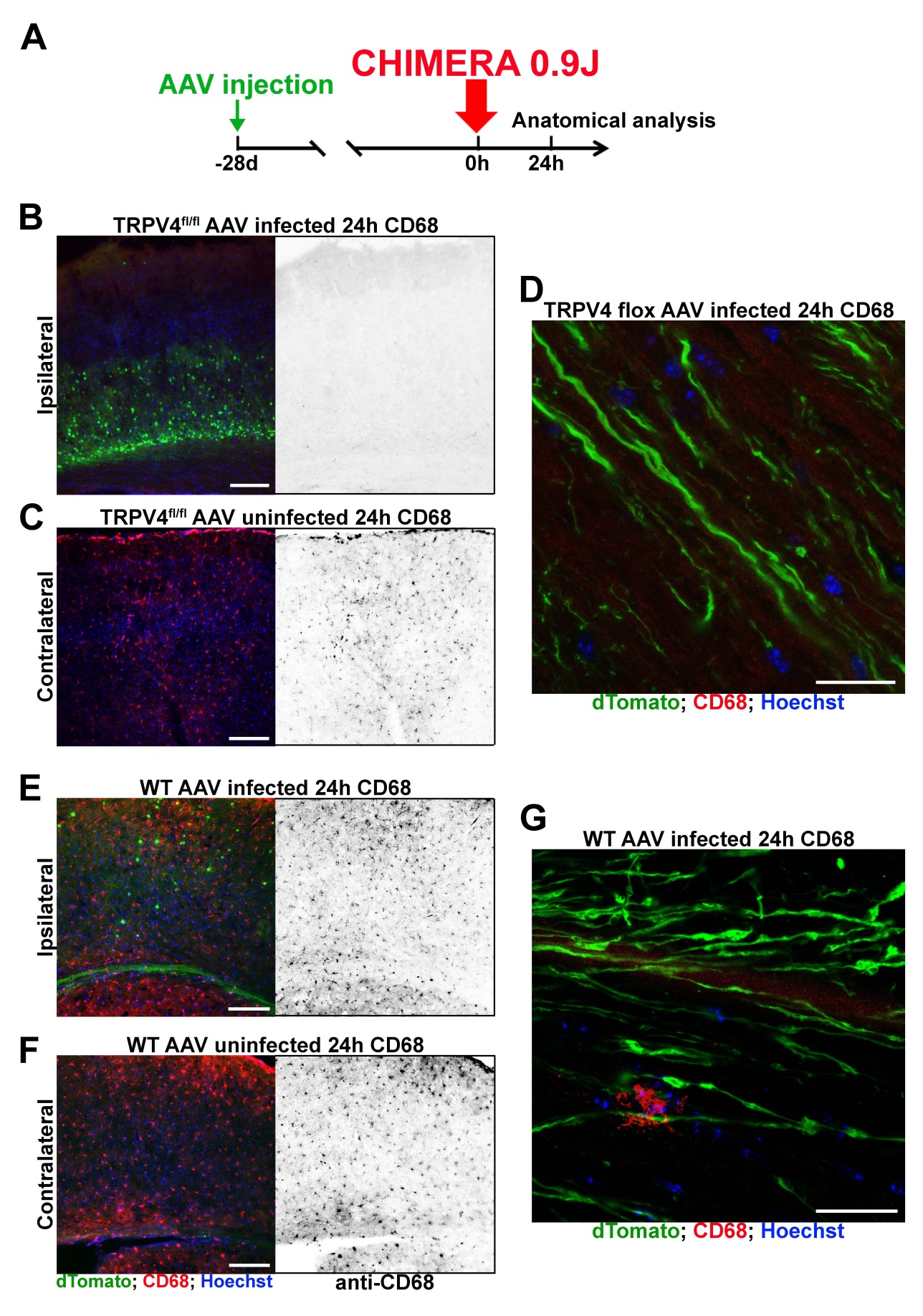
**

**Supplementary Fig. S9. Acute knockdown of TRPV4 in neurons significantly inhibited adjacent microglial activation induced by CHIMERA.**

(**A**) The experimental diagram. TRPV4^fl/fl^ (**B**)-(**D**) or WT (**E**)-(**G**) mice (2 months old) were injected with AAV9-Syn-Cre-dTomato. Four weeks (28d) later, the mice were impacted with CHIMERA (0.9J) and then perfused and fixed either immediately (0h) or 24 h later (24h).

Low magnification images of the Ipsilateral (**B**) and Contralateral (**C**) side of the TRPV4^fl/fl^ mouse cortex with dTomato in green and CD68 staining in red in merged images. The CD68 staining signals are inverted in gray-scale images.

(**D**) Confocal image of the Ipsilateral EC in (**B**).

Low magnification images of the Ipsilateral (**E**) and Contralateral (**F**) side of the WT cortex with dTomato in green and CD68 staining in red in merged images.

(**G**) Confocal image of the Ipsilateral EC in (**E**).

Scale bars, 300 μm in (**B**), (**C**), (**E**), and (**F**); 20 μm in (**D**) and (**G**).
